# Mechanism of mRNA-STAR domain interaction: Molecular dynamics simulations of Mammalian Quaking STAR protein

**DOI:** 10.1038/s41598-017-12930-2

**Published:** 2017-10-03

**Authors:** Monika Sharma, C. R. Anirudh

**Affiliations:** Department of Chemical Sciences, Indian Institute of Science Education and Research (IISER), Sector 81, Knowledge City, SAS Nagar, Punjab, India

## Abstract

STAR proteins are evolutionary conserved mRNA-binding proteins that post-transcriptionally regulate gene expression at all stages of RNA metabolism. These proteins possess conserved STAR domain that recognizes identical RNA regulatory elements as YUAAY. Recently reported crystal structures show that STAR domain is composed of N-terminal QUA1, K-homology domain (KH) and C-terminal QUA2, and mRNA binding is mediated by KH-QUA2 domain. Here, we present simulation studies done to investigate binding of mRNA to STAR protein, mammalian Quaking protein (QKI). We carried out conventional MD simulations of STAR domain in presence and absence of mRNA, and studied the impact of mRNA on the stability, dynamics and underlying allosteric mechanism of STAR domain. Our unbiased simulations results show that presence of mRNA stabilizes the overall STAR domain by reducing the structural deviations, correlating the ‘within-domain’ motions, and maintaining the native contacts information. Absence of mRNA not only influenced the essential modes of motion of STAR domain, but also affected the connectivity of networks within STAR domain. We further explored the dissociation of mRNA from STAR domain using umbrella sampling simulations, and the results suggest that mRNA binding to STAR domain occurs in multi-step: first conformational selection of mRNA backbone conformations, followed by induced fit mechanism as nucleobases interact with STAR domain.

## Introduction

STAR (Signal Transduction and Activation of Ribonucleic acid) family of proteins are mRNA-binding proteins linking signal transduction with post-transcriptional gene regulation at various levels, such as mRNA shuttling, pre-mRNA splicing, and all stages of RNA metabolism^1^. Well studied examples include Splicing Factor 1 (SF1) mediating the intron recognition during spliceosome assembly^2–4^, Src-associated during mitosis 68-kDa protein (SAM 68) regulating alternative splicing in response to extracellular cues^4–8^, and Quaking related (QR) proteins^9–12^, critical in modulating development processes such as mammalian spermatogenesis (mice QKI), metazoan central nervous system development (GLD-1 in worms)^13–16^, sperm-to-oogenesis in hermaphrodites (GLD-1)^17–19^, or Drosophila wing development (HOW in flies)^20–22^. In humans, QKI proteins have been reported to be associated with numerous human pathologies like cancers^12,23^ and neurological disorders such as human inherited ataxia, multiple sclerosis, or schizophrenia^24,25^. These recent findings warrant the need to understand the molecular basis of RNA recognition, binding, and regulation by STAR proteins.

**Figure 1:**
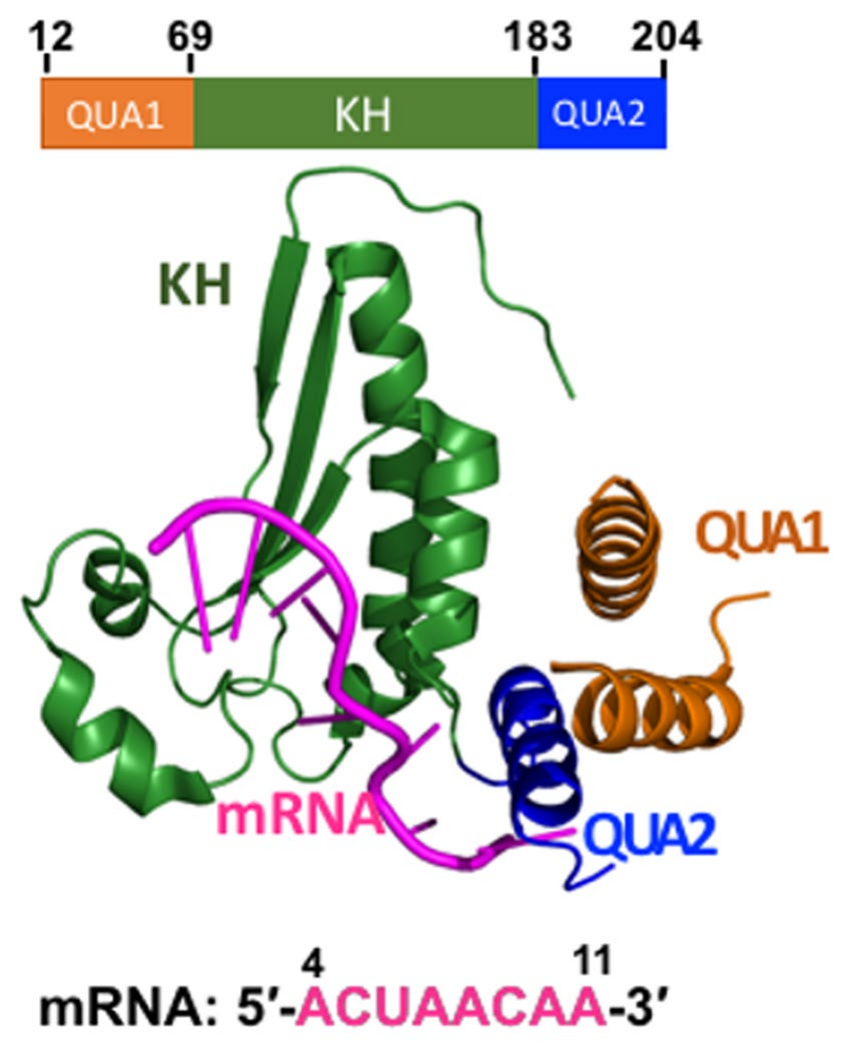
Crystal structure of monomer of QKI STAR-RNA complex (PDBid: 4JVH). Domain architecture of QKI domain is shown, consisting of N-terminal QUA1 domain (in orange), mRNA-binding KH-domain (in green), and C-terminal QUA2 domain (in blue). RNA target is shown in magenta.

Interestingly, all these proteins share a hallmark STAR domain of ~200 amino acids that recognizes and binds mRNA, and is conserved from yeast to humans^1,24^. STAR domain consists of N-terminal dimerization QUA1 domain, followed by mRNA binding extended single heterogeneous nuclear ribonucleoprotein K- Homology (KH) domain^3,7,26–28^. The latter is a conventional type I KH domain of β_1_α_1_α_2_β_2_β_3_α_3_ topology which recognizes four nucleotides of RNA by van der Waals forces, hydrophobic and electrostatic interactions^29^. Further downstream 2-3 nucleotides are recognized by fourth helix of C-terminal QUA2 domain^28^. Due to high sequence similarity, STAR family of proteins bind identical RNA regulatory elements (RREs) YUAAY irrespective of their regulatory consequences^28,30–32^. Structure of the intact QKI STAR domains in complex with *in vivo* high affinity RNA targets has recently been solved^28^, revealing relative arrangement of QUA1, KH, and QUA2 domains within each subunit of STAR homodimer. Both KH and QUA2 form an extended interface with bound mRNA (Fig. 1), and homodimerization is mediated exclusively by QUA1. Structure of STAR domain of mammalian Quaking protein in absence of RNA ligand is not available. However, structure of mRNA free KH-QUA2 region of *Xenopus* Quaking homologue, pXqua has been reported^33^. pXqua shows remarkable 94% sequence identity with human and mouse QKI proteins^34^. Based on ^15^N relaxation parameters of QUA2 region of pXqua2, Maguire *et al*.^33^ concluded that while KH domain is well defined, C-terminal QUA2 region in absence of RNA ligand is more dynamic and partially structured in absence of mRNA (Figure SI1A). Authors^33^ speculated that QUA2 forms rigid helical structure once cognate mRNA is docked onto KH-QUA2 domain. These structures have thus, provided valuable insights into the molecular recognition of mRNA by STAR domain. However, they ignore information regarding the dynamic behavior of these domains that play critical role in their structural stability, and are relevant to the recognition and binding of mRNA. In this study, we employed atomistic molecular dynamics (MD) simulations to derive this information by studying the dynamics of intermolecular interactions in mRNA bound complexes of STAR domains of QKI. MD studies have been reliably applied previously to complexes of RNA with U1A protein containing the most abundantly found RNA recognition motif^35–37^. Therefore, the current study also aims to expand our understanding of the recognition of RNA elements by the second most occurring recognition fold in protein, K homology fold in STAR proteins.

Here, MD simulations have been carried out for STAR domain of Quaking protein in presence and absence of bound mRNA. Since our aim is to investigate influence of mRNA binding onto STAR domain, we have ignored the information of N-terminal dimerization QUA1 domain, and considered only truncated KH-QUA2 domain, as the latter is solely responsible for mRNA binding. We then analyzed the modes of dynamics using principal component anlaysis within the simulated systems and identified allosteric communications within the networks created using contact information and correlation data. Later on, we employed umbrella sampling technique to investigate how mRNA unbinds from pocket of STAR domain.

## Materials and Methods

### Simulated systems and Simulation protocol

Molecular dynamics (MD) simulations of monomers of QKI with bound mRNA were carried out with explicit solvent. As our aim is to understand mRNA recognition and binding, we considered only mRNA binding KH-QUA2 domain for this study. Coordinates for mRNA bound KH-QUA2 domain of QKI were extracted from PDBid^28^: 4JVH (residues 69–204). In addition, RNA free conformations of QKI proteins were also simulated by removing mRNA coordinates from these mRNA-bound complexes. Herein, we refer to mRNA bound KH-QUA2 domain as HOLO state, and artificially generated mRNA free KH-QUA2 structure as APO state.

The CHARMM36 force field with the CMAP correction^38^ was used to describe molecular interactions. All simulations were run with NAMD2.11^39^. The complexes were solvated in a rectangular TIP3P solvent box^40^ and neutralized with Na^+^ ions^41^, resulting in simulation box size of 84Åx83Åx84Å consisting ~60,000 atoms. Additional Na^+^ and Cl^−^ ions were added at a concentration of 0.15 M NaCl salt. Long-range electrostatic interactions were treated using the Particle Mesh Ewald (PME) technique^42^ as implemented in NAMD^43^. A non-bonded cut-off of 12 Å was applied to the Lennard-Jones potential and a cut-off for the direct-space part of the Coulomb forces with a switching function starting at distance 10 Å and reaching zero at 12 Å. A 2 fs time step was used for all MD simulations in combination with SHAKE^44^ to holonomically constrain bonds involving hydrogens. Rigid water molecules were constrained using the SETTLE algorithm^45^. The systems were minimized under harmonic restraints of 2 kcal/mol/Å^2^ in three steps. Initially, the backbone atoms of the protein and RNA were restrained while allowing only the water molecules to reorient for 50,000 steps. Then, restraints on only Cα atoms of protein were applied to allow the protein and RNA to relax during an additional 50,000 steps of minimization. Finally, the restraints were reduced to 1 kcal/mol/Å^2^ and the structures were minimized further for another 50,000 steps. The minimized structures were heated gradually from 0 K to 300 K at a rate of 30 K/60 ps while still maintaining the restraints. The simulation at 300 K was then continued for 100 ps before gradually releasing the restraints from 1 kcal/mol/Å^2^ to 0 during another 200 ps. The final structures were then equilibrated for 10 ns under NPT ensemble. The simulation temperature and pressure was maintained at 300 K and 1 atm using Langevin dynamics^46^ and Nose-Hoover Langevin piston method^47,48^, respectively. After equilibration, structures were subjected to NPT simulations. This method was repeated four times with different initial velocity assignments using random seed method to generate four independent production runs. The four runs span different simulation times of 75 ns, 100 ns, 200 ns, and 250 ns; reaching a total simulation time of 0.625 µs for mRNA bound complexes. Similarly, mRNA free APO proteins were simulated three times generating three independent production runs spanning simulation times of 100 ns, 150 ns and 250 ns; resulting in total simulation time of 0.5 µs.

### Analysis of the trajectories

Trajectory analysis or in particular, structure analyses involving the deviations (RMSD, RMSF), intramolecular contacts, native contacts within RNA binding KH domain, protein-mRNA contacts, were carried out using VMD. RMSD and RMSF calculations have been done with reference to crystal structure, and for conformations sampled at every 2 ps during runs. Structural convergence of different runs was assessed based on computation of root mean square average correlation (RAC) of protein KH-QUA2 domain at different time intervals using the *rmscorr* command^49^ in CPPTRAJ^50^. For RAC calculation, the fit was done over Cα atoms of running average structures of residues 69–204 over the full trajectories calculated at each time interval from 0 ps to 250 ns referenced in the RMS fit to the average structure over the entire trajectory run. For evaluation of native contacts, we calculated for two measures: Q, the fraction of native contacts and Qs, similarity of natively contacting residue pairs to their native distances using carma software^51^. For Q and Qs calculation, a native contact is defined when Cα atom of one residue interacts with Cα of another residue (excluding the adjacent two neighboring residues) within cutoff distance of 8.0 Å. Qs is similar to Q except that a Gaussian penalty is introduced if the native contact is far from its native distance^52^. Native contacts between KH-QUA2 domain were calculated for KH-QUA2 domain after pooling in all the conformations sampled at every 2 ps during different trajectory runs. Variations in interactions between mRNA and protein residues within conformation sampled at every 2 ps were analyzed using VMD for different trajectory runs of HOLO state.

Principal component analysis has been done with Bio3D package written in R^53,54^. A variance-covariance matrix was constructed using Cα atoms after pooling in all the conformations (sampled every ps) from the trajectories, and time-dependent projections of the first three principle components onto the trajectories have been studied. Contact map was constructed for Cα atom of crystal structure using Bio3D package, considering contacts between cutoff of 8 Å, excluding neighboring residue.

### Determination of allosteric communications

Allosteric networks within the proteins were identified using the NetworkView plugin^55,56^ of VMD. The dynamical networks were constructed using data from all the trajectories of the protein-RNA complexes sampled every 2 ps. For each molecular system, a network graph was constructed with two nodes for each nucleotide molecule, N1 and N9 atoms were used to define the nodes for the base atoms for pyrimidines and purines, respectively; and phosphorus, P atom was used to define the nodes for the sugar and backbone phosphate atoms. To define nodes for each protein residue, Cα atoms were used. All the conformations were pooled in to calculate the local-contact matrix, where a contact was defined between the two nodes (excluding the neighboring nodes) within 4.5 Å, and observed for at least 75% of the simulation time. The so-constructed contact matrix was then weighed by the correlation values of the two end nodes, where C_ij_ is the correlation matrix calculated as ${C}_{ij}={\langle {\rm{\Delta }}{{r}}_{{i}}\cdot {\rm{\Delta }}{{r}}_{{j}}\rangle /\langle {\rm{\Delta }}{{r}}_{i}^{2}\rangle }^{1/2}{\langle {\rm{\Delta }}{{r}}_{j}^{2}\rangle }^{1/2}$. The correlation matrices were calculated using carma software^51^, and the shortest paths between two nodes in the network were found using the Floyd-Warshall algorithm^57^. Characteristic path lengths (CPL), average of all the shortest paths, were calculated as indicative of size of networks for both HOLO and APO states. The community structure was identified by using Girvan-Newman algorithm^58^, which uses edge betweenness to detect community peripheries. Betweenness of an edge is defined as the number of shortest paths between pairs of nodes that run along it, and is a measure of influence of a node over the flow of information between other nodes. Community detection is a iterative process, where each cycle consists of calculation of betweenness of all edges in the network, followed by removal of edge with the highest betweenness until no edge remain.

### Umbrella sampling simulations

The limitation with the conventional MD is that the simulated systems get easily trapped in their local minima, and thus, cannot be used for sampling conformational transition processes such as the unbinding or binding of ligands. We employed umbrella sampling technique^59–61^ to investigate the unbinding of mRNA from STAR domain. In order to simulate the unbinding process, distance between the centers of mass defined by group of atoms of STAR domain as well as for mRNA was used as reaction coordinate (RC). For mRNA, the P and O5’ atoms of nucleic backbone of nucleotides 5 to 9 were used to calculate the center of mass. For STAR domain, center of mass was calculated for group of Cα atoms present within 5.0 Å of mRNA in crystal structure. The RC was extended from initial distance of 10.5 Å to 20 Å (Figure SI2). Umbrella sampling run consisted 15 simulation windows with 0.5 Å in length for RC between 10.5 Å to 15 Å; and 1 Å for RC between 15 Å to 20 Å. Here an elastic constant of 5 kcal/molÅ^2^ was used in all the US simulation windows. For each window, 50 ns were performed to converge the sampling, thus approximate 750 ns US simulations have been performed for the complex.

The aim of this study is to investigate the dissociation of mRNA from STAR domain, and not to calculate the binding free energy. Thus, we did not use the earlier proposed constrained scheme for ligand unbinding^62–64^. Nevertheless, to prevent the drifting of the systems, especially for STAR domain, two restraints were applied. First, orientation restraint of 100 kcal/mol Å^2^ was added to the Cα atoms of the protein to tackle the rotational drift. Secondly, restraint of 10 kcal/mol Å^2^ was added to the Cα atoms that are 15 Å away from the mRNA in the crystal structure to restrain them to the center of mass as calculated for similar atoms in the crystal structure. Potential of mean force (PMF) was constructed along the RC using Weighted Histogram Analysis Method (WHAM)^65,66^, after discarding the first 5 ns as equilibration period. Error bars were calculated with Monte Carlo bootstrap error analysis. RC was separated into 1000 bins for the WHAM calculation, with iteration tolerance value set to 0.0001. Temperature was set to 300 K to keep consistence with the simulation temperature.

It is possible to estimate multidimensional PMFs along additional degrees of freedom, whether biased or unbiased, with the assumption that all other degrees of freedom orthogonal to the biasing coordinates were thoroughly sampled^67^. To investigate the energy profile as mRNA explored different conformations within each US window, we calculated two-dimensional PMF along the rmsd of backbone atoms of mRNA as additional degree of freedom and biased RC. The rmsd values were calculated with respect to crystal structure for backbone atoms of nucleotides 5 to 10 (excluding O1P and O2P oxygen atoms of phosphate backbone moiety) for conformation sampled within each window, and these additional coordinates were added to the biasing coordinates with zero force constant. For obtaining two-dimensional PMF profile along RC and rmsd, the data was binned into 500 bins for 2D-WHAM calculation with tolerance value of 0.0001 and temperature set to 300 K.

Backbone conformations sampled by mRNA within each window during umbrella sampling was quantified in detail using two measures: sugar puckering configurations and pseudo-torsion angles. These calculations were performed for mRNA conformation sampled using 3DNA software^68^. The backbone pseudo-torsion angles^69^ were defined as: η: C4′(i-1)-P(i)-C4′(i)-P(i + 1) and θ: P(i)-C4′(i)-P(i + 1)-C4′(i + 1).

## Results

We have carried out simulations of mRNA bound and mRNA free extended KH (KH-QUA2) domains of QKI proteins. Throughout this manuscript, for convenience and clarity, we will refer these mRNA bound structures as HOLO states and mRNA free structures as APO states.

### Stability of STAR domain

**Figure 2:**
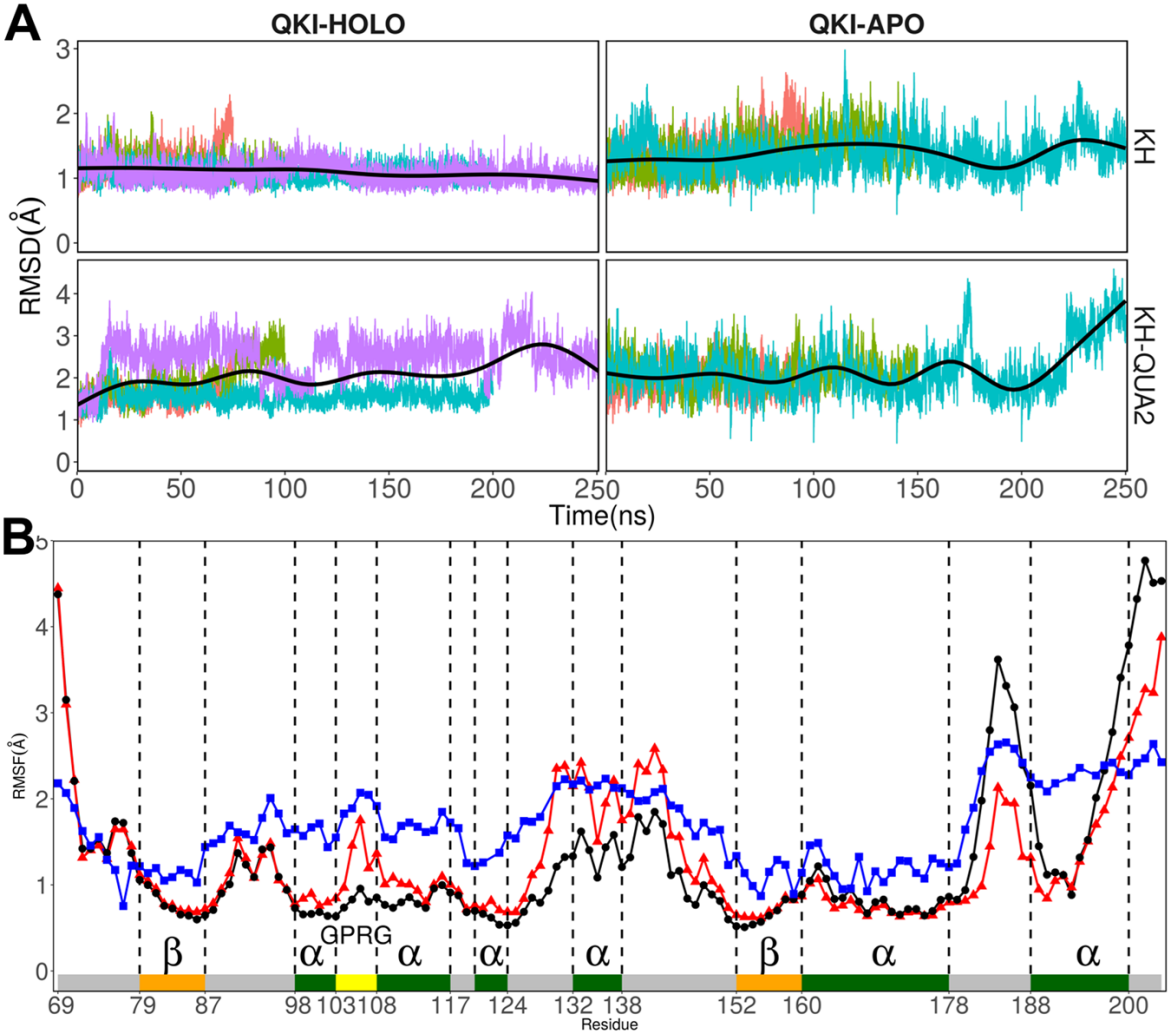
(**A**) Time-evolution of RMSD of STAR domain of QKI protein in presence of mRNA (QKI-HOLO state) and in absence of mRNA (QKI-APO state). RMSD values during independent runs are shown in varied colors, with averages shown in black thick line. (**B**) RMSF for C atoms of KH-QUA2 domain of QKI protein averaged over all the simulation runs. Data with black circles and black lines is for HOLO state, red colored triangles with red lines is for APO state, and blue colored squares with blues lines correspond to converted B-factors for crystal structure (4JVH). Secondary structure elements: alpha helices, beta sheets and loops are annotated with boxes of green color, orange color and grey color. ‘GPRG’ motif is annotated with yellow colored box.

We analyzed changes in root mean square deviations (RMSD) values of the protein backbone atoms of QKI domain with respect to the crystal structure coordinates in response to the presence or absence of mRNA. Figure 2A shows the time evolution of RMSD values of backbone atoms of truncated KH-QUA2 protein by using crystal structure as reference for each simulation run. On an average, RMS deviations are lower for mRNA bound HOLO structures compared to mRNA free APO structures. To further investigate which part of STAR domain is contributing to large RMSD values, we carried out domain-wise RMSD calculations of KH and QUA2 domains. The time evolution plots of RMSDs of KH domain during each simulation run (Fig. 2A) indicate that mRNA binding stabilizes the KH domain and present low RMSD values in HOLO states. Inclusion of helical C-terminal QUA2 domain increases the RMSD values for both HOLO and APO states, indicating high flexibility of C-terminal domain irrespective of presence of mRNA.

The root mean square fluctuations (rmsf) is another indicator of the flexibility of certain residue during simulations. We calculated rmsf values of Cα atoms of each protein residue, averaged over all the conformations sampled during different independent simulations runs for both HOLO and APO structures. For comparison with crystal structure, we converted B-factors or temperature factors to rmsf values by using Debye-Waller formula $({RMSF}={(\frac{3B}{8{\pi }^{2}})}^{1/2})$ Figure 2B shows that in presence of mRNA, KH domain in HOLO state shows less fluctuations than observed in APO states. mRNA is bound onto hydrophobic surface created by the residues from helices α3, α4, strand β2, conserved GPRG, and loops connecting β2 and α5 of KH domain. Although these residues show high mobility in crystal, the rmsf values are lower in HOLO state than in APO state. The residues at the loops and at both N- and C-terminal regions are flexible in both mRNA bound and free states. It is noteworthy that residues in the C-terminal QUA2 region show elevated experimental RMSF, and exhibit large RMSF values in both APO and HOLO states, indicating that QUA2 domain is still relatively flexible in solution irrespective of presence of mRNA. The higher mobility of QUA2 domain is further confirmed by the comparison with its higher rms deviations observed for both APO and HOLO states.

We further assessed the convergence of each simulation run by computing pseudo-autocorrelation functions for RMSD values: root mean square (RMS) average correlation (RAC). A converged trajectory is when slope of RAC values approaches zero, indicating no further changes in the average structure as sampling time increases^49^. For QKI system, we fit each trajectory to the overall average structure and observed that for both HOLO and APO states: as simulation time increases, RAC slope smoothly decreases nearing zero at 250 ns (Figure SI3). However, at this point we will not exclude the possibility that time scales may remain shorter to observe any drastic changes that would have occurred over longer timescales and reflected in the RAC values.

Comparing mRNA free APO state simulation results with experimentally available pXqua KH-QUA2 domain structure, we observe that though KH-QUA2 domain is well defined in both proteins, QUA2 domain is more flexible in pXqua protein. Solution structure of mRNA-free APO form of pXqua KH-QUA2^33^ shows significant flexibility in C-terminal QUA2 domain with disordered helical structure and possesses different orientations with respect to α6 of KH domain (Figure SI1A). It should be noted here that pXqua QUA2 domain is larger in size (26 residues: 188–214) compared to QKI QUA2 domain (16 residues: 188–204). In addition, overall secondary structure assignment of solution structure of mRNA free pXqua KH-QUA2 is considerably different from X-ray determined structure of QKI KH-QUA2 (Figure SI1B). This may be the reason that we do not see drastic changes in our QKI-APO state simulations, besides the limitations in timescales of conventional MD simulations.

The fact that Maguire *et al*. speculated^33^ that QUA2 domain ought to be preformed even in absence of ligand in order to have effective mRNA binding; and since no experimental structural information is available for either mRNA bound pXqua KH-QUA2 or mRNA free QKI KH-QUA2 domain, it remains unclear about the exact conformational transition of KH-QUA2 domain going from ligand free APO state to mRNA bound HOLO state.

### Correlations within STAR domain

**Figure 3:**
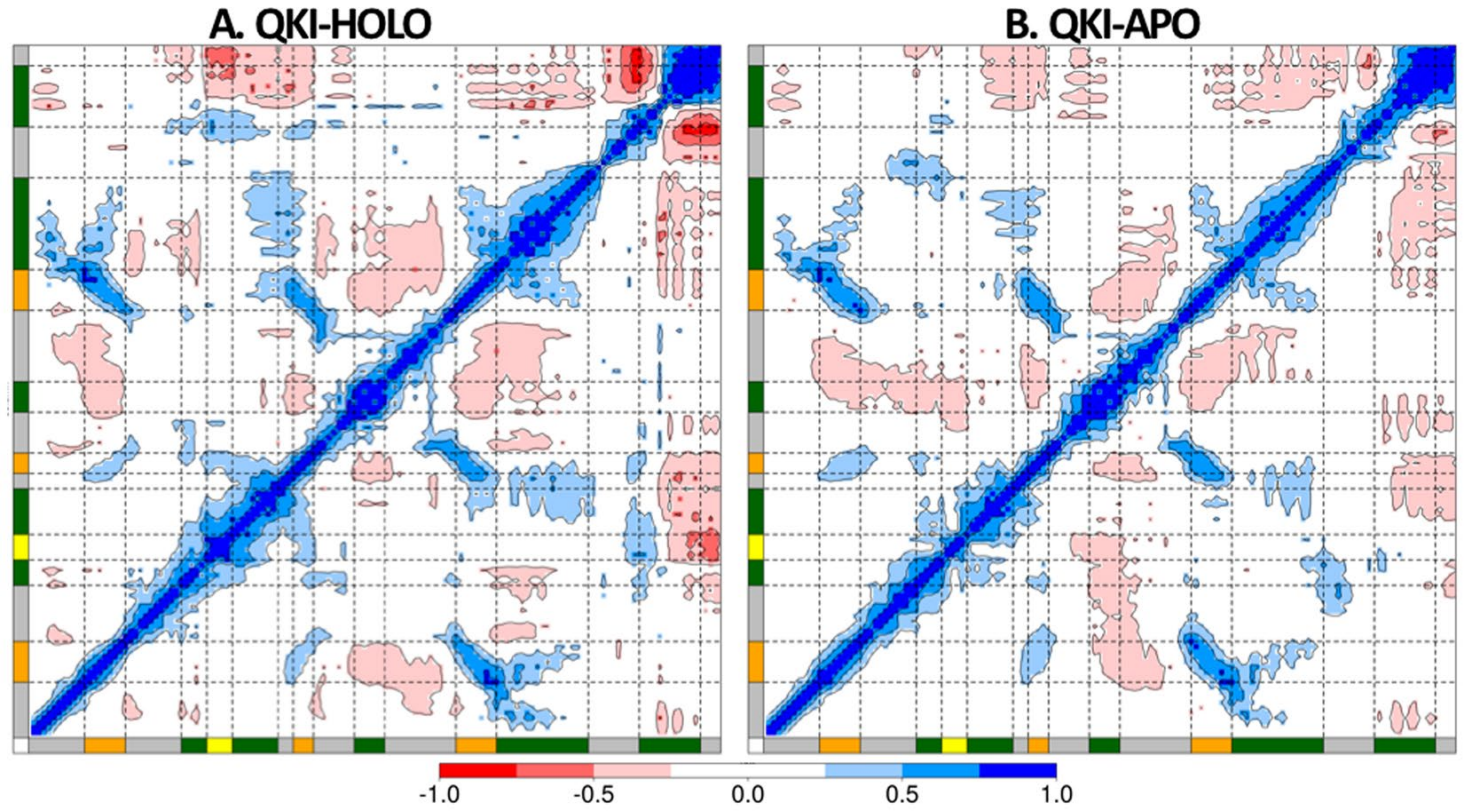
Dynamic cross correlation maps calculated as time-averaged for Cα atoms of KH-QUA2 domain of QKI protein (**A**) in presence of bound mRNA, QKI-HOLO and (**B**) in absence of mRNA, QKI-APO. Secondary structure elements are shown as in Fig. 2.

We calculated the correlation matrix for Cα position fluctuations of QKI for HOLO and APO states. Dynamic cross-correlation maps for the two states, representing correlation coefficients calculated as averaged over all the conformations sampled during the simulations, are shown in Fig. 3. The whole range of correlation from −1 to + 1 is represented in three ranges: blue color corresponding to positive correlation values ranging from 0.25 to 1; red color corresponding to negative correlation values ranging from −0.25 to −1; and white color corresponding to weak or no-correlation values ranging from −0.25 to + 0.25. The extent of correlation or anti-correlation is indicated by variation in the intensity of respective blue or red color. A quick glance at the maps indicates that there is loss of correlation when mRNA is not bound as shown by reduction in colored islands in DCC maps of APO vs HOLO. Comparing with the contact map constructed from crystal structure coordinates of QKI STAR domain (pdbID: 4JVH) (Figure SI4), we see that residues that form contact within crystal structure are positively correlated in both HOLO and APO states.

In QKI, mRNA binds onto KH domain within the cleft formed by helices α3, α4, α6, and strands β1, β2, β3 on one side and α5 on other side. The binding is further extended by helix α7 of QUA2 domain. mRNA specifically interacts with the residues from helices α3, α4, strand β2, conserved GPRG, and loops connecting β2 and α5 of KH domain. These residues are observed to be highly self-correlated as indicated by the blue-colored islands in DCC map of HOLO state. This self-correlation decreases in absence of bound mRNA as observed from DCC map of APO state. Interestingly, looking at the correlation between two sides of cleft of mRNA binding region of KH domain, we observe that in presence of mRNA, correlation of α5 on one side of the cleft with mRNA-binding helices α3 and α4 on other side of cleft is lost. α5 is anti-correlated with helix α6 and strands β1, β2, and β3 in HOLO state. In APO state, as mRNA is absent, though α5 remains anti-correlated with the strands β1, β2, and β3 on the other side of cleft, it loses the correlation with helix α6 and gets anti-correlated with mRNA-binding helices α3 and α4 on other side of cleft.

In presence of mRNA, motion of helix α7 of C-terminal QUA2 domain is anti-correlated with all the structural elements of KH domain, except β1 and α5. In absence of mRNA, helix α7 gets anti-correlated with α5, and for the other SSEs, the extent of anti-correlation reduces as observed by reduction in the size as well as intensity of red colored islands. The generated DCC maps for both HOLO and APO states thus, disclosed that mRNA binding influences the nature of correlated motions of STAR domain.

### Impact of mRNA on the interactions within STAR domain

**Figure 4:**
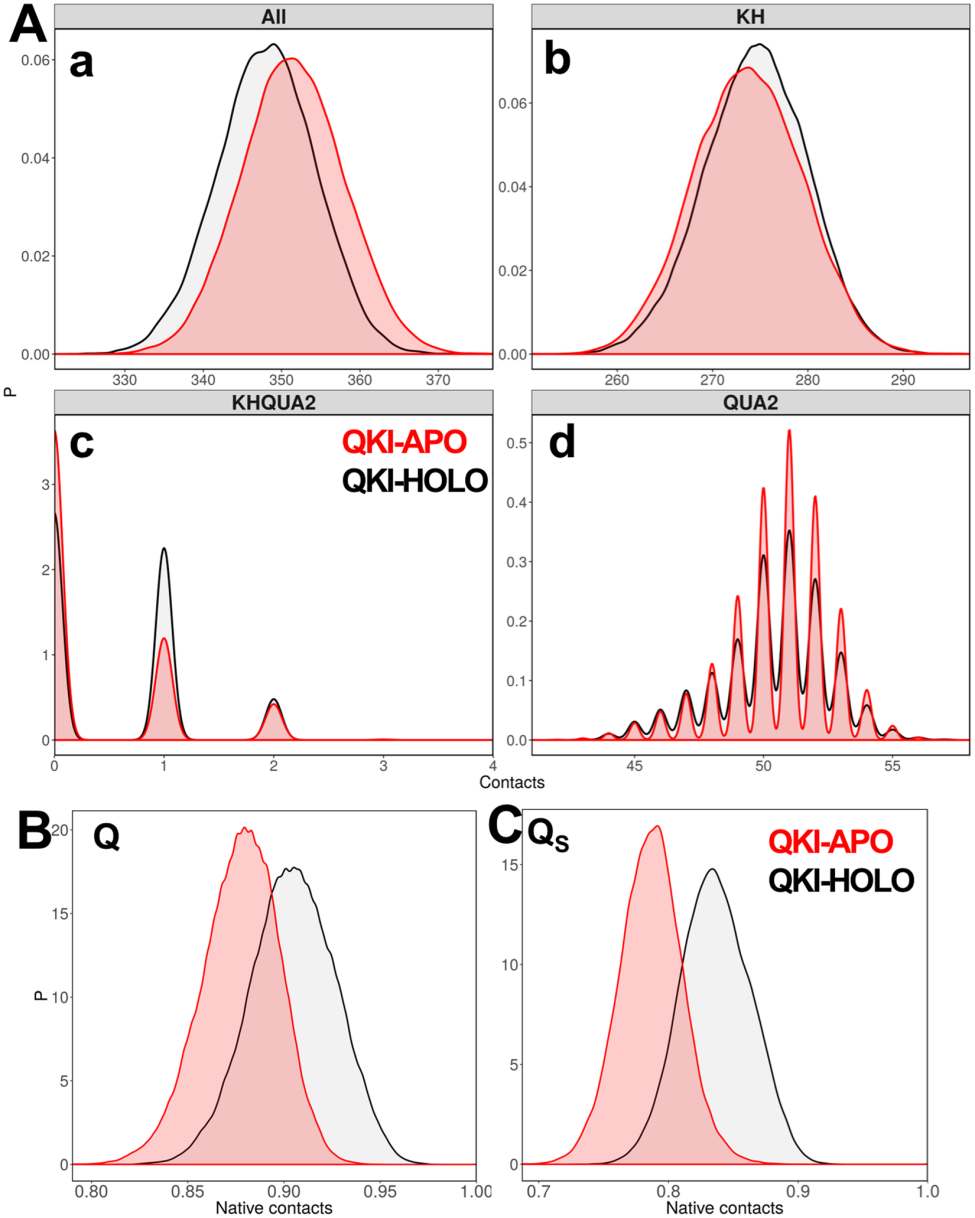
(**A**) Distribution of native contacts within STAR domain of QKI protein during the simulations (a) within maxi KH (KH-QUA2) domain, (b) within KH domain only, (c) between KH domain and QUA2 domain, and (d) within QUA2 domain. Probability distribution functions for (**B**) fraction of native contacts, Q and (**C**) similarity of natively contacting residue pairs to their native distances, Q_s_ for both HOLO (in black) and APO (in red) states.

To assess the impact of mRNA on the intra-residual contacts within STAR domain, we calculated total contacts for conformations sampled during each simulation run. Whenever one Cα atom sees another Cα atom within 6.0 Å, we define it as a contact. The distribution plots for the contacts (Fig. 4A) for the truncated KH-QUA2 domain suggest that in absence of mRNA, mRNA binding maxi-KH domain has more intramolecular contacts, which reduces in presence of mRNA. We further breakdown these contacts as intramolecular contacts within KH domain, within QUA2 domain, and inter-domain contacts between KH and QUA2. Interestingly, we observed that while for KH domain, the intramolecular domain contacts are slightly more in HOLO state than in APO state; the increase in total number of contacts in STAR domain is in fact consequence of increase in (a) intramolecular contacts within QUA2 domain and (b) interdomain contacts of QUA2 with KH domain. This suggests that in absence of mRNA, protein residues are free to form another set of interactions and inter-domain interactions between KH and QUA2 increases. Now, the question arises about the fate of native contacts that are present in experimental crystal structures; and how important is the presence of mRNA for intramolecular contacts within STAR domain.

To answer this query and further evaluate the structural differences in STAR domain associated with mRNA binding with respect to the experimental crystal structure, we calculated the fraction of native contacts (Q) and the similarity of natively contacting residue pairs to their native distances (Q_S_) for each conformation sampled during all the simulation runs for HOLO and APO states. Plotting the probability distribution functions for Q (Fig. 4B) and Q_S_ (Fig. 4C) for both HOLO and APO states, we observe that absence of mRNA shifts the peak plot to the left indicating loss of native contacts in APO state. The similarity measure, Q_S_ not only takes into consideration the existence of contact, but also quantify how close the contact is to the native distance^52^. Thus, the differences in HOLO and APO state peaks for Q_S_ in comparison to Q are modest yet subtle. The contact analyses indicate that though in absence of mRNA, STAR domain forms more number of intramolecular contacts; presence of mRNA is essential for maintaining the native contacts within STAR domain.

### Relative motions of STAR domain in response to mRNA

**Figure 5:**
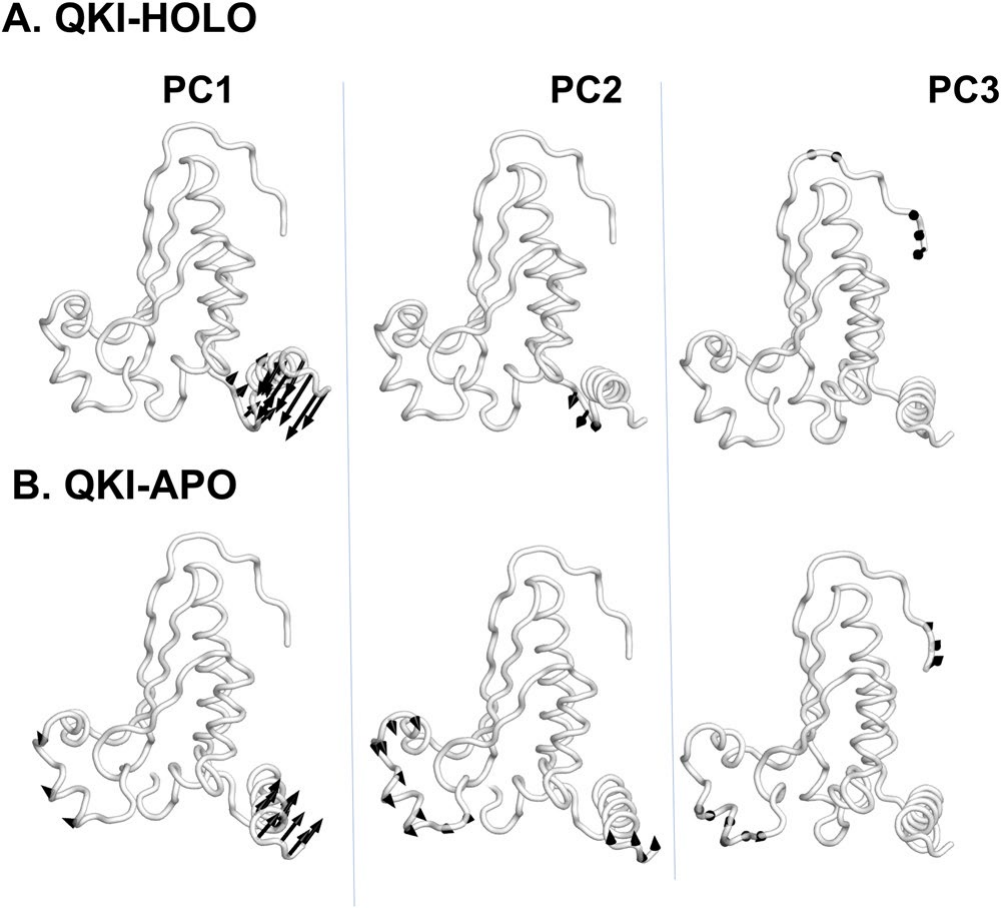
Motions along the first three principal components are shown for (**A**) mRNA bound HOLO and (**B**) mRNA free APO state.

Principal component analysis (PCA) separates conformational space of a system into essential subspaces by filtering global slow motions from the fast motions^70^. Thus, majority of the dynamics can be described by a surprisingly low number of collective degrees of freedom. Figure SI5 shows that eigenvalues for both HOLO and APO states drop steeply in the first ten eigenmodes; and the detailed information related of the top three principal movements (PC1, PC2, and PC3) is presented in Fig. 5. The pointing of the arrows shows the direction of the motion vector and, the length of arrows represents the motion magnitude. We observe that STAR domain conducts differential motion modes in presence or absence of bound mRNA. In mRNA bound HOLO state, first principal mode is dominated by hinge movements in only QUA2 domain with stable KH domain. In APO state, when mRNA is not bound movements are observed in helix α5 and QUA2 domain. For QUA2 domain, the direction of the movements in APO seems to be opposite to those observed in HOLO state. In second and third modes of HOLO state, movements are observed for flexible KH-QUA2 linker, and N-terminal of KH domain, respectively. For APO state, both second mode and third mode is dominated with correlated movements of helix α5 with C-terminal of QUA2 domain and N-terminal of KH domain, respectively. The results of PCA analysis are in consistent with the DCCM analysis that motion of α5 of KH domain is negatively correlated with motion of α7 of QUA2 domain as suggested by their movements in opposite directions in the first mode of APO state.

### Network Analysis of STAR domain in response to mRNA

**Figure 6:**
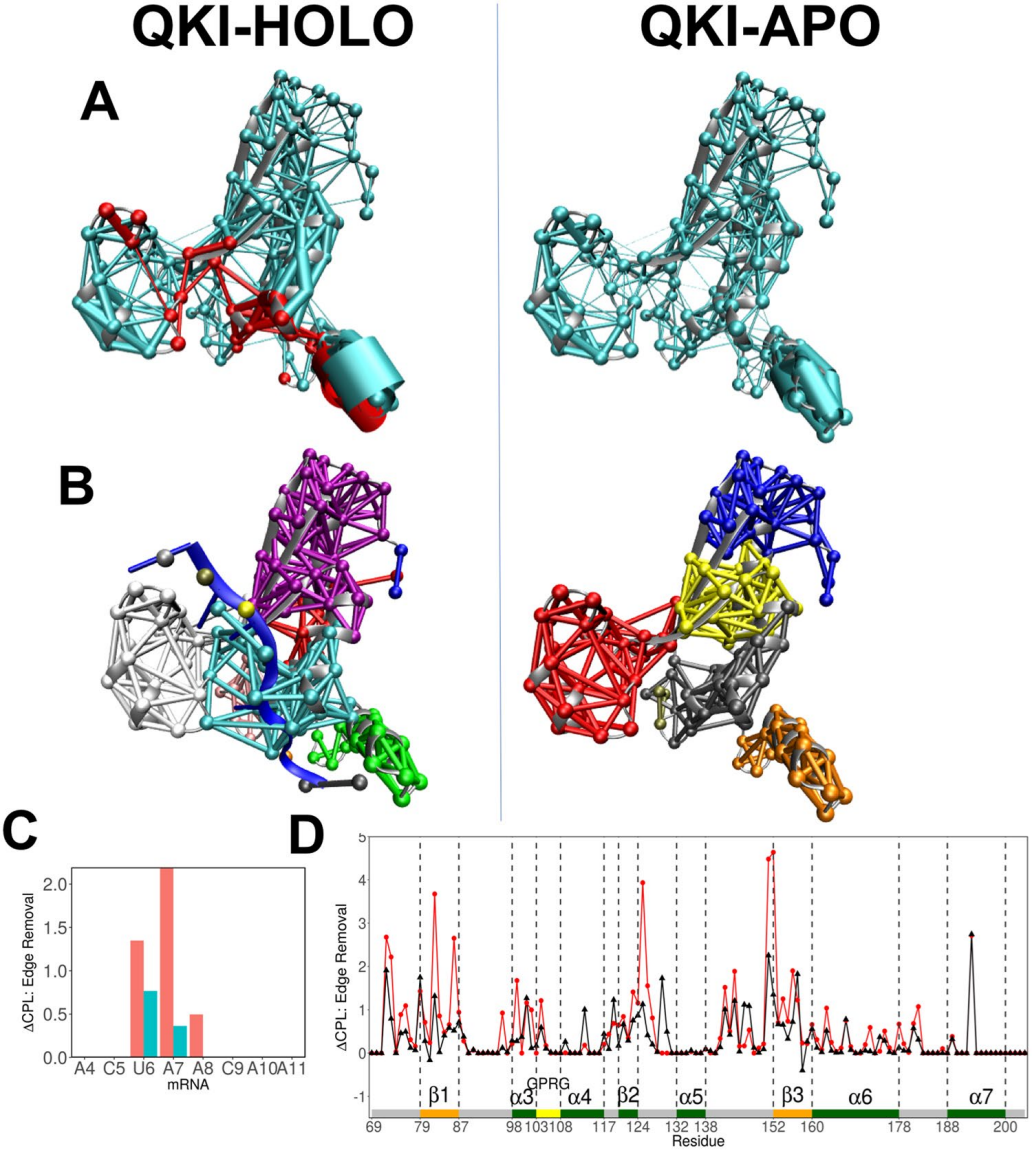
(**A**) Networks, weighed by correlation data, within KH-QUA2 domain of QKI protein is shown for mRNA-bound HOLO state and mRNA free APO state. Red colored nodes and edges correspond to interactions within 8 Å of mRNA. (**B**) Splitting of the networks into different communities are shown. (**C**) Change in CPL upon edge removal of nucleotide is shown for mRNA. Red and blue colored bars correspond to two nodes representing nucleotide base and sugar-phosphate backbone, respectively. (**D**) Change in CPL upon edge removal shown for KH-QUA2 domain for mRNA bound HOLO state (black colored) and mRNA free APO state (red colored). Structural elements are depicted as in Fig. 2B.

We combined the correlation data obtained from MD simulations with the structure-based network analysis to determine evolution of residue interaction networks in presence or absence of mRNA. Protein structure networks were constructed by incorporating the topology-based residue connectivity in combination with the contact maps of residue cross-correlations obtained from MD simulations. Each residue and nucleotide represents a node in the network, and two nodes are connected by an edge if (a) nodes are non-neighbors, and (b) they are in contact within 4.5 Å of each other for more that 75% of simulation time. The significance of node is determined further by the dynamic information flow through that edge as measured by the correlation between respective nodes. The extent of correlation between the two nodes is depicted by the width of the edge, with wider edges indicating strongly correlated nodes. The weighted dynamical network representations for HOLO and APO states in Fig. 6A depicted the changes in interactions among residues in response to mRNA. The differences in networks are significantly visible in the mRNA binding cleft region of STAR domain as nodes are observed to have wider edges for helices α5, α6, α7 (QUA2 domain) and strand β3, suggesting enhanced stability and correlation within the STAR domain in presence of mRNA. Similar trend is observed in DCCM analysis discussed above.

We analyzed the variations in the connectivity of networks in response to mRNA by viewing the changes in formation of local communities (Fig. 6B). Nodes within one community are more strongly interconnected to each other, compared to nodes within other communities. The way Girvan-Newman algorithm splits the HOLO and APO states is suggestive of the influence of mRNA binding to the STAR domain. In APO state, distinct communities are formed around mRNA binding cleft region: (a) N-terminal upper right part of cleft (β1, β2, α4), (b) middle right part of cleft (β3, α3), (c) left part of cleft (α5), (d) down right of cleft (α6), and (e) QUA2 domain (α7). For HOLO state, the arrangement of communities changes and major communities observed are: (a) upper right and middle of cleft region merges as one community, (b) interface mRNA nucleotides and their protein interaction partners, (c) left part of cleft (α5), and (d) QUA2 domain (α7). Interestingly, residues from α3 (on right side of cleft) and α5 (on left side of cleft) form one community along with bound mRNA in HOLO state. In APO state, these form distinct communities.

To further identify nucleotides involved in communication across the interface, we looked at the differences in characteristic path length (CPL) upon edge removal for each nucleotide (Fig. 6C). Sethi *et al*.^54^ observed that for allosteric signal transmission in the aaRS:tRNA complex, nucleotides involved in communication significantly increased the edge CPL. Similarly, for STAR-mRNA complex, nucleotides that significantly increased the edge CPL are the ones forming direct interactions with STAR domain: U6, A7, and A8. These residues form part of invariant core sequence observed via genome-wide search of STAR binding element of QKI: NA(A > C)U(A≫C)A(underlined nucleotides correspond to U6, A7 and A8)^31,32^. The stronger correlation of these nucleotides with STAR domain is also reflected in their interaction analyses. These three nucleotides form consistent native interactions with their interaction partners in STAR domain during all simulation runs compared to other nucleotides in the mRNA element (Figure SI6).

We also analyzed variations in CPL upon complete removal of nodes (Figure SI7) and edges (Fig. 6D) for STAR domain for HOLO and APO states. Though the results of node removal are a bit noisy, edge removal results seem to be more informative. The nucleotide binding residues in STAR domain are observed to increase CPL not only in HOLO state, but also in APO state. A noteworthy comparison is the alone increase in CPL upon removal of Q193 for QUA2 domain irrespective of presence of mRNA. Q193 is involved in interaction with U6 of mRNA, and this interaction remains consistent throughout the simulations of HOLO state.

### Mapping simulation data with mutational data

Deleterious effects have been observed for N-ethyl-N-nitrosourea (ENU)-induced hydrophobic-to-polar point mutation, V157E in KH domain of QKI^24,71^. Another hydrophobic-to-polar mutation I155N showed severe developmental defects in zebrafish^72^. We analyzed solvent accessible surface area (SASA) values for these residues and observed average values as: I155 (0.06 ± 0.29), V157 (5.93 ± 3.97) for HOLO state, and I155 (0.06 ± 0.25), V157 (5.06 ± 4.05) for APO state simulations. Lower SASA values observed for these residues irrespective of mRNA presence indicate that these residues are buried within the hydrophobic pocket, and suggest that their mutation to polar residues will affect mRNA binding by strongly impairing the protein stability.

In homologous GLD-1 protein, eight missense mutations abolishing essential GLD-1 function in the germline have been identified: G200E, P217L, G227D, G248R, G250R, A294T, G308E, and D310N^19^. In QKI, these residues are equivalent to: G77, P94, G104, G125, G127, A171, G185, and D187, respectively. Teplova *et al*.^28^ investigated *in vivo* and *in vitro* RNA-binding affinities with the mutations of key amino acids within the KH and QUA2 domains of QKI contacting specific bases of mRNA. Three double-mutants were identified: K190A/Q193A, N97A/R130A, and K120A/R124A^28^. As measured by ITC experiments K190A/Q193A mutation resulted in 2-fold reduction in *in vitro* RRE binding affinities, N97A/R130A mutation drops affinity by 10-fold, and K120A/R124A by 20-fold. Similar trend is observed for *in vivo* RRE binding quantified with PAR-CLIP. The signal for K190A/Q193A was reduced 5-fold, the signal for N97A/R130A was reduced 10-fold, and the signal for K120A/R124A was reduced 20-fold compared with wild-type protein.

**Figure 7:**
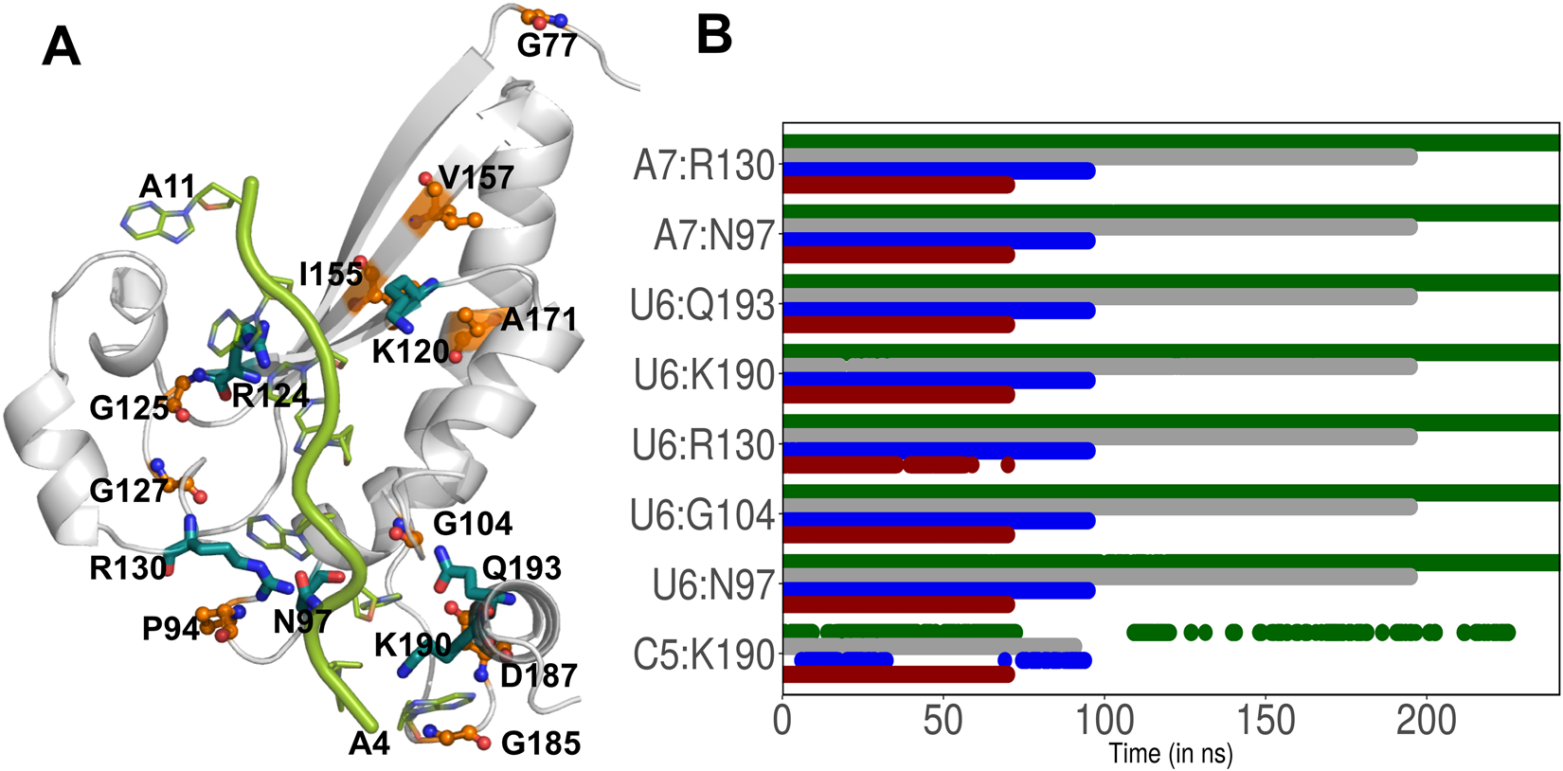
(**A**) Spatial organization of various mutations (reported for STAR proteins) in QKI protein. (**B**) Time evolution plots depicting interactions between mRNA nucleotide and its amino acid partner in STAR domain. Here interactions present in crystal structure are considered. Different colors indicate four different runs for HOLO state.

Spatial organization of these residues in the bound mRNA-STAR domain complex indicates (Fig. 7A) that probable effect of mutations of residues: N97, G104, K120, R124, G125, R130, K190, and Q193 is because of their role in interacting mRNA nucleotides. Looking at the time evolution plots of native mRNA-protein interaction analysis (Fig. 7B), we observed that K190 interacts with C5 and U6 for more than 75% of simulation time. The interactions of U6 with N97, G104, R130 and Q193 are consistent throughout the simulations. U6 forms hydrogen bonds with G104 and Q193. Similarly, interactions of A7 with N97 and R130 are consistent throughout the simulations. As per deviation analysis, elevated rmsf values are observed for N97, G104, K120, R124, G125, R130 and Q193 residues in mRNA free APO states in comparison with bound HOLO states. Interestingly, some of these residues also show increase in characteristic path length for both APO and HOLO states, indicating their importance in functioning of STAR domain. Eight missense equivalent residues in QKI show comparative large increase in ∆CPL upon node removal in APO state than in comparison with HOLO state, corroborating their role in structural stability of STAR domain (Table SI1). Residues that interact with mRNA show increase in ∆CPL upon node removal in HOLO state than in APO state indicating their role in recognition and binding of mRNA (Table SI1).

### Dissociation of mRNA from bound STAR complex

**Figure 8:**
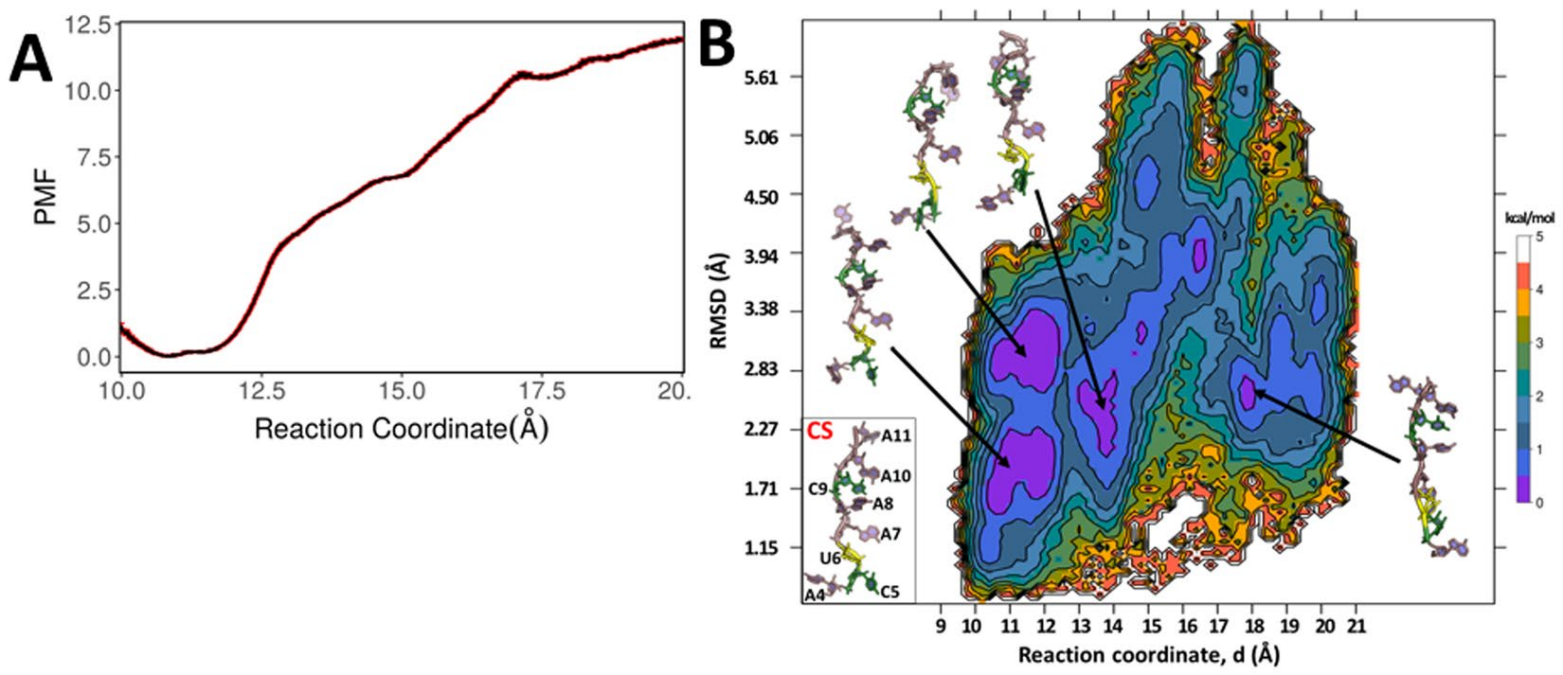
(**A**) 1-D Potential of mean force (PMF) profile along the biased reaction coordinate as obtained from 15 umbrella sampling simulations within range of 10.5–20 Å. Errors in the PMF profile are shown in red. (**B**) Two-dimensional free energy profile along the biased reaction coordinate, d and rmsd values determined from umbrella sampling simulations. The representative structures are shown for each minima observed by violet-colored islands. Crystal structure of bound mRNA is shown for comparison.

Conventional MD simulations have their limitations when it comes to simulate transition processes involving higher energetic barriers. For instance, simulation of dissociation of mRNA from bound STAR domain may take even more than millisecond of level of conventional simulations. To overcome this problem, we carried out umbrella sampling simulations to investigate unbinding process of mRNA from KH-QUA2 domain of QKI protein. We considered reaction coordinate as separation distance of mRNA from QKI domain, and from initial value of 10.5 Å, the distance was extended to 20 Å. We calculated PMF as a function of this reaction coordinate to determine the energetics of dissociation of mRNA from bound QKI-STAR complex (Fig. 8A). The comparative analysis of PMF profile indicates that dissociation of mRNA is energetically unfavorable process, and PMF values are lower for separation distance between 10.6–11.5 Å. This corresponds to the range observed for RC during conventional MD of bound HOLO state. As distance is decreased beyond this range, the energies rise resulting in dissociation of mRNA from the complex.

As we investigate further the conformational sampling of mRNA along the RC, we calculated two-dimensional PMF along biased reaction coordinate and unbiased coordinate as rmsd of mRNA with respect to crystal structure (Fig. 8B). We observe that as the mRNA dissociates from the complex, it has larger rmsd values from the initial structure. Interestingly, for the favorable RC range of 10.6–11.5 Å, we observed two minima at rmsd ranges of 1.7–2.2 Å and 2.8–3.3 Å as indicated by violet-colored islands in two-dimensional plot. mRNA within this RC range show lesser rmsd values than 4 Å as it is still interacting with protein. However, moving along RC beyond, we do observe violet colored minima at RC range of 13–14 Å with rmsd values of 1.8–2.6 Å. Few scattered lower energy conformations with rmsd less than 3 Å are observed at higher RC values. This suggests that even in absence of STAR domain, backbone of single stranded mRNA is able to adopt conformations similar to crystal structure. Looking at the representative structures for minima, we find that backbone conformations are similar to that observed in crystal structure but nucleobases adopt different conformations.

For such a small mRNA, RMSD values of more than 2 Å may include diverse backbone conformations. Thus, we further assessed the nucleic backbone conformations by calculating sugar puckering angles (Figure SI8) and pseudo-torsions (Figure SI9) for the nucleotides within each simulation window. In crystal structure, ribose sugars of nucleotides 4–7 are C2′-*endo* and 8–11 are C3′-*endo*. Looking at the puckering behavior of these residues, we observe that as mRNA dissociates from complex, the nucleotides 4–7 show transitions from C2′-*endo* to C3′-*endo* and occasionally to O4′-*endo* as well. However, nucleotides 8–11 remain primarily as C3′-*endo*. This similar behavior is reflected in behavior of pseudo-torsions, where η-θ plots are analyzed and compared with crystal structure bound mRNA (Figure SI9). We analyzed the time-dependent variations of pseudo-torsions as dial plots (Figure SI10) where variations of η and θ for each nucleotide of mRNA conformation sampled within each simulation window is shown. Mapping the pseudo-torsion plots with 2D PMF plot, we observe that mRNA backbone comprising nucleotides U6, A7, A8, C9, and A10 show similar patterns at low and higher RC values and these may contribute to violet colored minima at higher RC values. For instance, conformations sampled at RC 10.5 Å for backbone variations of U6, A7, A8, C9, and A10 are visited at RC value of 14–14.5 Å, or even at 17–19 Å.

These analyses suggest that conformation selection should be primary binding mechanism for mRNA to be recognized by STAR domain, and nucleobases rearrange later in vicinity of protein to interact with amino acid partners, probably via induced fit mechanism.

## Discussion

In this study, we have carried out simulations of mRNA bound and mRNA free truncated maxi-KH (KH-QUA2) domain of STAR mammalian QKI protein. mRNA binds to KH domain by interacting with residues from helices α3, α4, strand β2, conserved GPRG, and loops connecting β2 and α5 of KH domain. The binding is further extended by helix α7 of QUA2 domain. Presence of mRNA lowers the deviations and fluctuations for the interacting regions within KH domain. Single helical QUA2 domain on the other hand is quite flexible irrespective of mRNA presence. Presence of mRNA influences the correlated motions within STAR domain as self-correlation within these binding regions decreases or changes in absence of bound mRNA. The underline reason behind this is attributed to variations in intra-residual contacts in KH-QUA2 domain in presence or absence of mRNA. Total number of intramolecular contacts within KH-QUA2 domain increases in absence of mRNA, though number of contacts within KH domain are less in APO state than in HOLO state. In APO state, flexible C-terminal QUA2 domain is free to have more interactions within itself and with mRNA-free KH domain. On the contrary, native contacts are maintained in presence of bound mRNA throughout the simulations of HOLO state. The first principal mode detected with PCA of APO state showed opening motions for mRNA binding cleft in absence of mRNA, indicating flexibility of KH-QUA2 domain where mRNA bind onto.

We further analyzed the KH-QUA2 domain in absence and presence of mRNA as network of interactions between the residues, with residues defined as nodes and interactions within them as edges. These edges were weighed with correlation data, and stronger correlations are observed in presence of mRNA. We further divided the network into local communities, and observed distinct communities in HOLO and APO states. C-terminal QUA2 domain in both HOLO and APO state forms one distinct community. More communities are observed in mRNA binding region of KH domain in APO state than in HOLO state. We computed differences in characteristic path length upon removal of each node or edge in the network. Noteworthy, we found that if we remove nodes or edges consisting nucleotides or residues that are experimentally reported significant for binding and regulation of mRNA by STAR domain in QKI or related STAR proteins, considerable differences in characteristic path length are observed.

We further investigated dissociation of mRNA from KH-QUA2 domain using enhanced umbrella sampling simulations. PMF profile calculated along biased reaction coordinate suggested that mRNA dissociation is an energetic unfavorable process, with minima near the bound values of complex. We further explored PMF along rmsd of mRNA with respect to the crystal structure as additional degree of freedom, and observed that even at higher RC values (indicating dissociated mRNA from STAR domain), few minima at lower rmsd values are observed. Mapping mRNA conformations to such regions, we observed that mRNA is able to adopt backbone conformations in absence of bound protein but the nucleobases adopt different conformations. This was further substantiated with calculation of sugar puckering angles and pseudotorsions to define backbone conformations of mRNA sampled within each umbrella sampling simulation window.

Based on our analysis on conventional and umbrella sampling simulations, we propose that mRNA binding is multi-step process. In APO state, STAR domain is flexible around mRNA binding cleft region formed by helix α5 on left side of cleft, and rest of KH and C-terminal QUA2 domain on right side of cleft. mRNA in free state is able to adopt multiple conformations, out of which extended backbone conformations similar to crystal structure are binding competent forms. Such conformations successfully interact onto the cleft region, and the nucleotides rearrange to form specific interactions with STAR domain via induced fit mechanism. A multi-step binding mode has very well observed in other biological complex systems^73–77^ as well. In order to delineate exact mechanism of mRNA binding, more extensive simulation studies are required which are beyond the scope of this research and is a subject of further interest.

## Conclusion

By using conventional simulations, we have investigated the influence of mRNA binding onto the KH-QUA2 domain of STAR Quaking (QKI) protein. The effect of mRNA binding has been analyzed on the basis of deviations, correlations, intramolecular contacts, and interaction network. Our results mapped with the experimental mutational data reported. We further explored the dissociation of mRNA from STAR domain by employing enhanced umbrella sampling techniques and we observed that binding of mRNA onto STAR domain is an interplay of conformational selection of extended mRNA backbone conformations followed by induced fit of nucleobases to their amino acid partners in STAR domain.

## Electronic supplementary material

Supplementary File
