# Supplementary material for: Mechanism of mRNA-STAR domain interaction: Molecular dynamics simulations of Mammalian Quaking STAR protein
Source: Sci Rep. 2017 Oct 3;7:12567. doi: 10.1038/s41598-017-12930-2 (PMC5626755; doi:10.1038/s41598-017-12930-2)
Supplement: Supplementary file 1 — Supplementary File [file 41598_2017_12930_MOESM1_ESM.doc]

**Mechanism of mRNA-STAR domain interaction: Molecular dynamics simulations of Mammalian Quaking STAR protein.**

Monika Sharma*, Anirudh Chandukudlu Rohithaswa

**Supplementary information:**


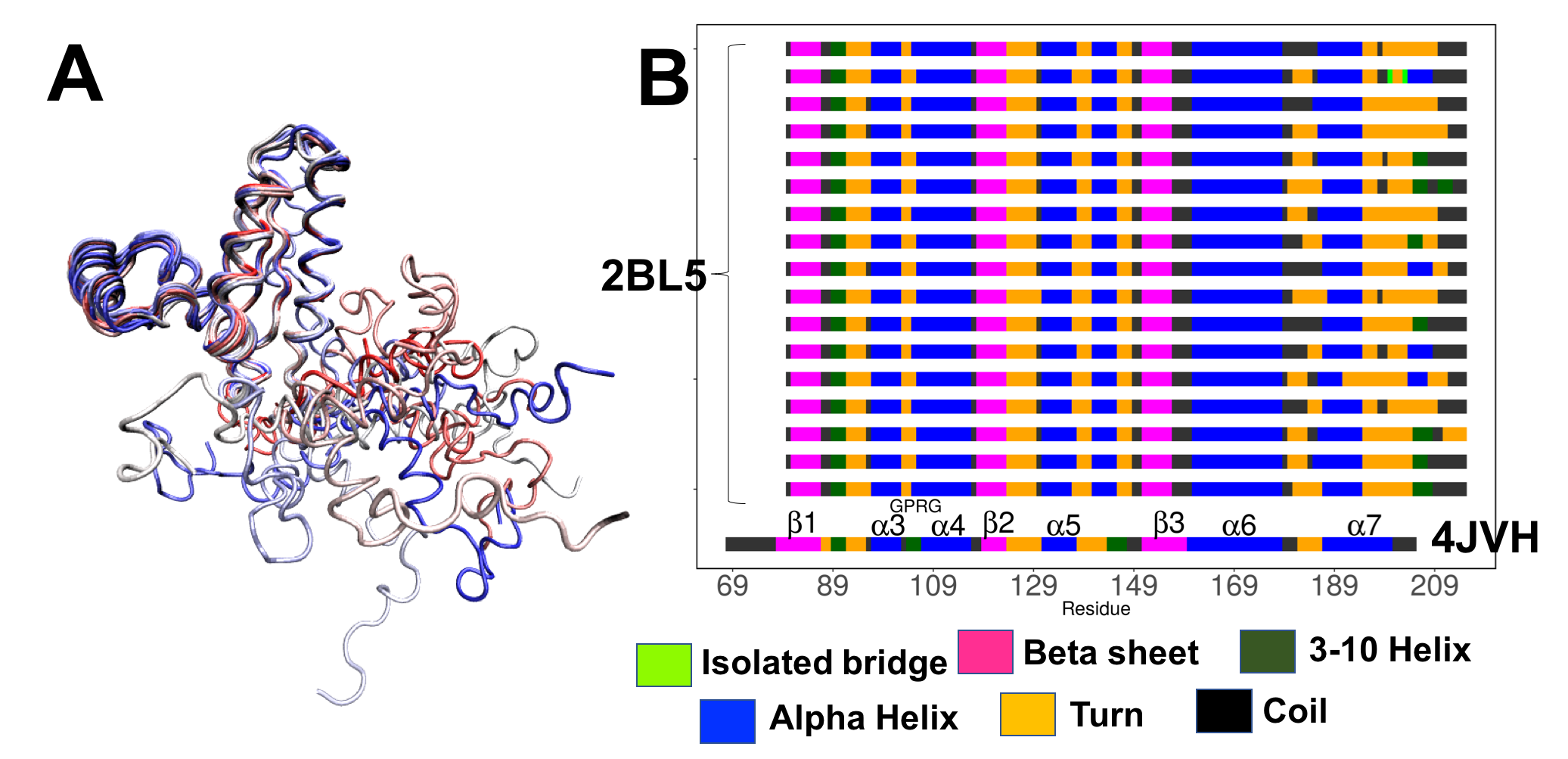


Figure SI1: A. Superposition of backbone of ensemble of solution structures of pXqua (PDBid: 2BL5). B. Secondary structure assignment for each of the solution structures of KH-QUA2 domain of pXqua using STRIDE software. In addition, structure assignment of crystal structure of QK-I KH-QUA2 domain is also shown with annotated secondary structure elements.


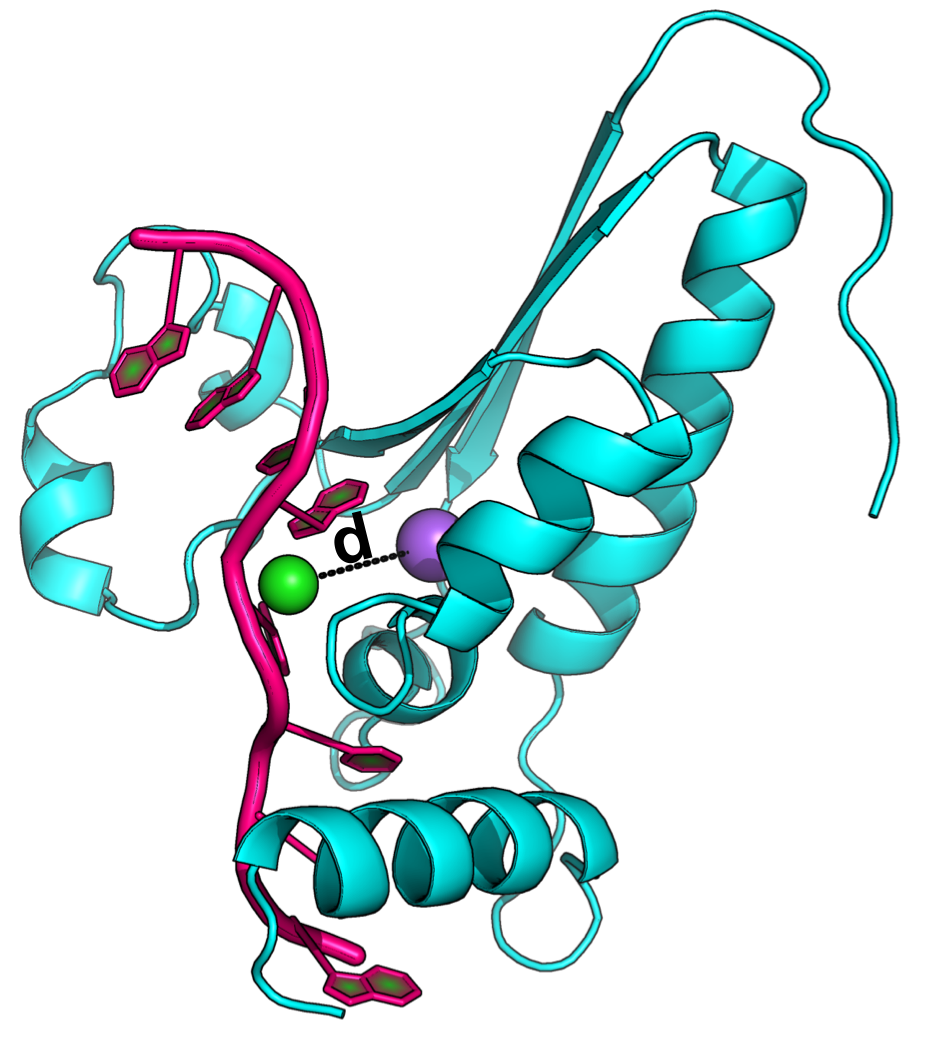


Figure SI2: Reaction coordinate (RC), d: distance between the centers of mass of STAR domain and mRNA used as reaction coordinate in umbrella sampling simulations. mRNA is shown in magenta and protein in cyan.


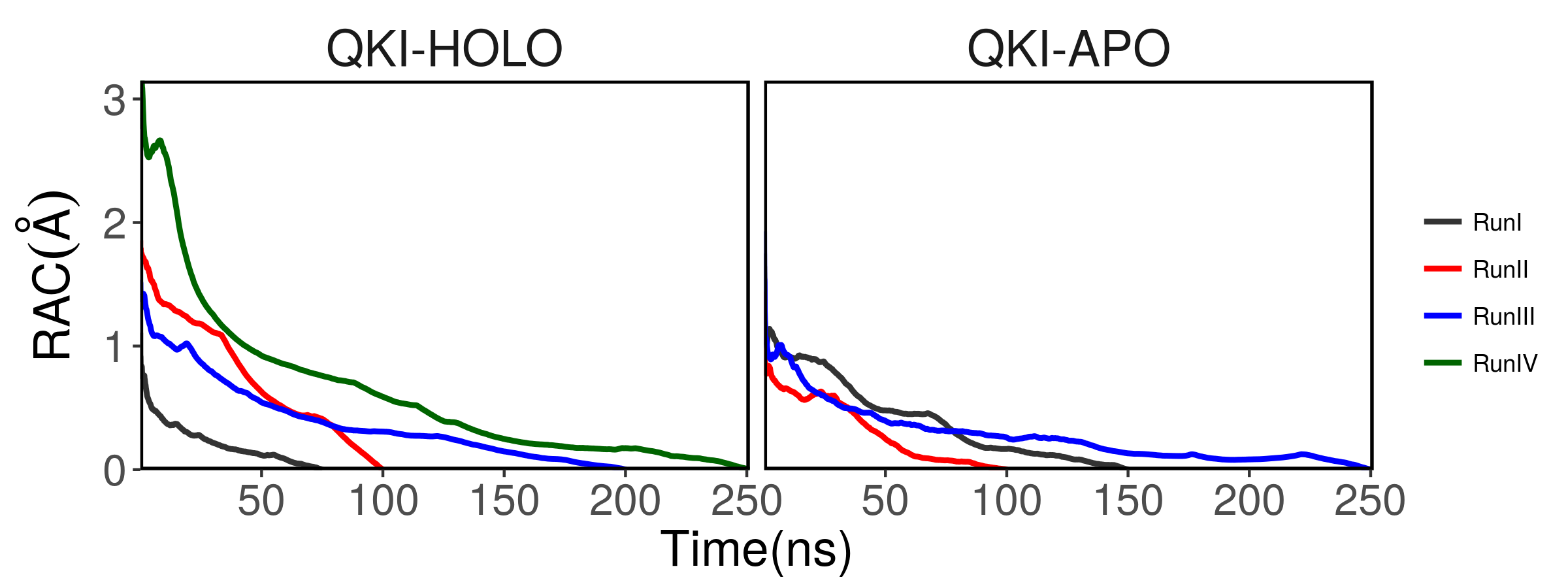


Figure SI3: RMS average correlation (RAC) computed at different time intervals over different runs, with fit over Cα atoms of KH-QUA2 domain with respect to the average structure over the entire trajectory.


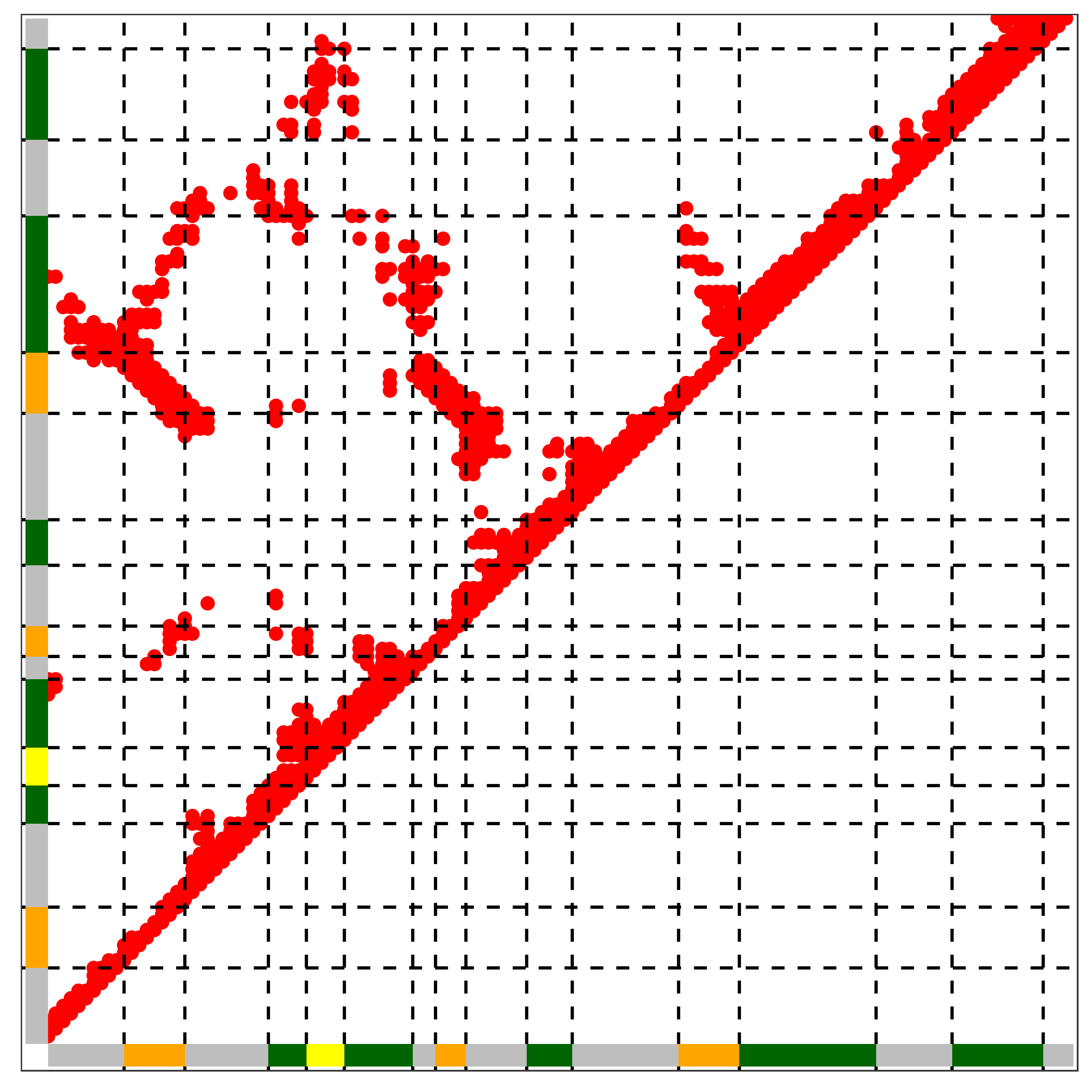


Figure SI4: Contact map calculated for Cα atoms of KH-QUA2 domain of crystal structure of QKI protein. (pdbid: 4JVH). Secondary structure elements are shown as in Figure 2.


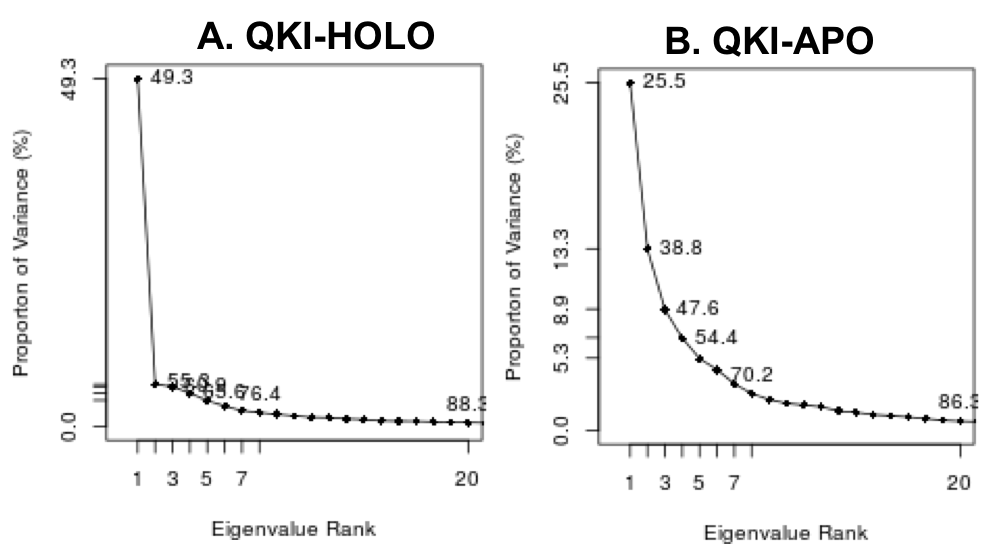


Figure SI5: Plot of eigenvalues of the covariance matrix versus the eigenvector index for A. mRNA bound HOLO state, and B. mRNA free APO state of QKI protein.


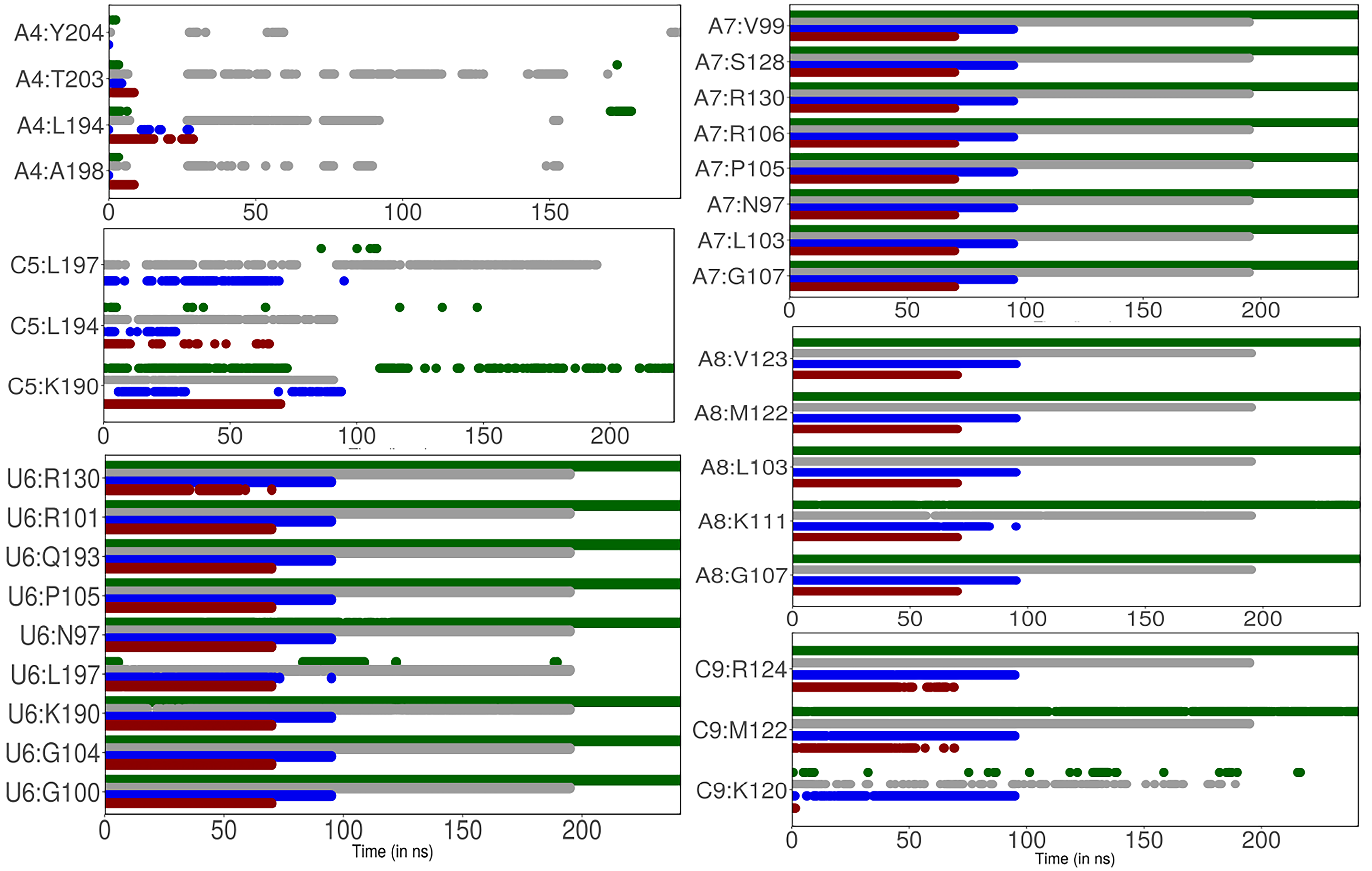


Figure SI6: Native state (as present in crystal structure) interactions between mRNA nucleotides and STAR domain are shown during time course of different simulation runs.


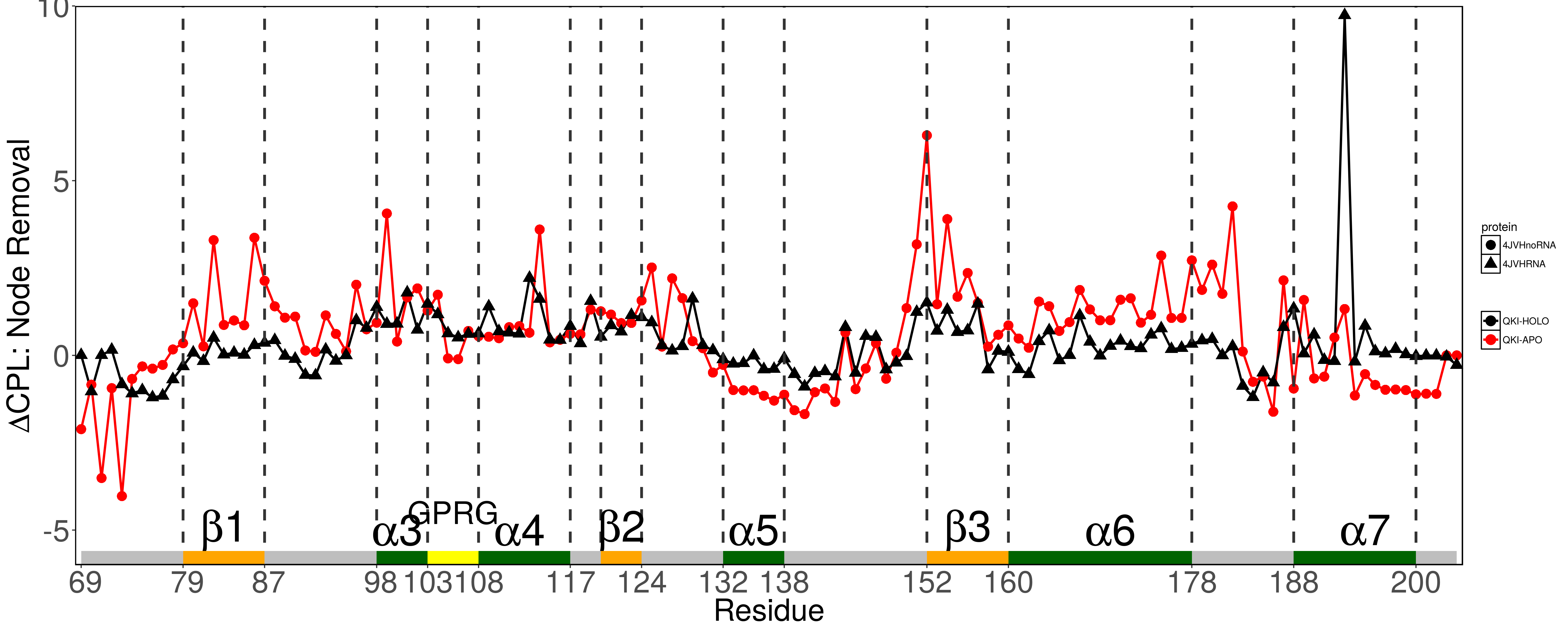


Figure SI7: Change in CPL upon node removal shown for KH-QUA2 domain for mRNA bound HOLO state (black colored) and mRNA free APO state (red colored). Structural elements are depicted as in Figure 2B.

Table SI1: Table showing changes in CPL (∆CPL) upon node and edge removal for HOLO and APO state of QKI protein

| Residue | ∆CPL: Node removal | | ∆CPL: Edge removal | |
| --- | --- | --- | --- | --- |
|  | APO | HOLO | APO | HOLO |
| G77 | -0.27 | -1.15 | 0.31 | 0.12 |
| P94 | 0.61 | -0.15 | 0.00 | 0.00 |
| N97 | 0.74 | 0.78 | 0.00 | 0.02 |
| G104 | 1.73 | 1.17 | 1.21 | 0.59 |
| K120 | 1.26 | 1.62 | 0.64 | 0.17 |
| R124 | 1.57 | 1.09 | 1.15 | 0.87 |
| G125 | 2.51 | 0.94 | 3.93 | 1.12 |
| G127 | 2.20 | 0.13 | 0.81 | 0.20 |
| R130 | 0.20 | 0.29 | 0.00 | 0.49 |
| I155 | 1.67 | 0.66 | 0.73 | 0.32 |
| V157 | 1.50 | 1.47 | 1.22 | 0.72 |
| A171 | 1.59 | 0.42 | 0.19 | 0.05 |
| G185 | -0.60 | -0.48 | 0.00 | 0.00 |
| D187 | 2.14 | 0.80 | 0.10 | 0.00 |
| K190 | -0.66 | 0.59 | 0.00 | 0.00 |
| Q193 | 1.32 | 9.73 | 2.71 | 2.74 |


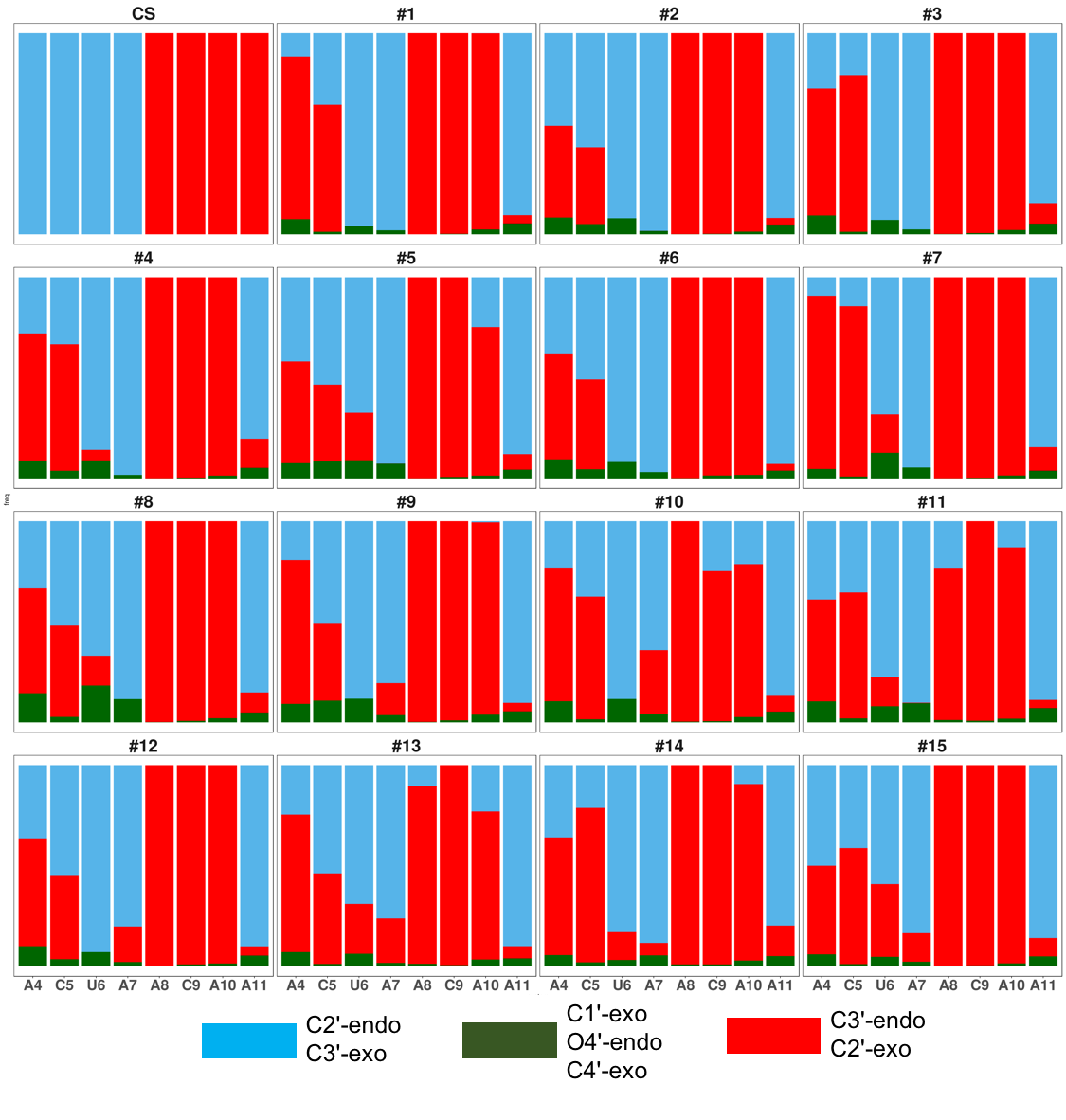


Figure SI8: Sugar puckering conformations observed for each nucleotide during simulations within all umbrella sampling windows as indicated by #N. The first panel (CS) indicates sugar puckering conformation for mRNA bound to the crystal structure (pdbid: 4JVH).


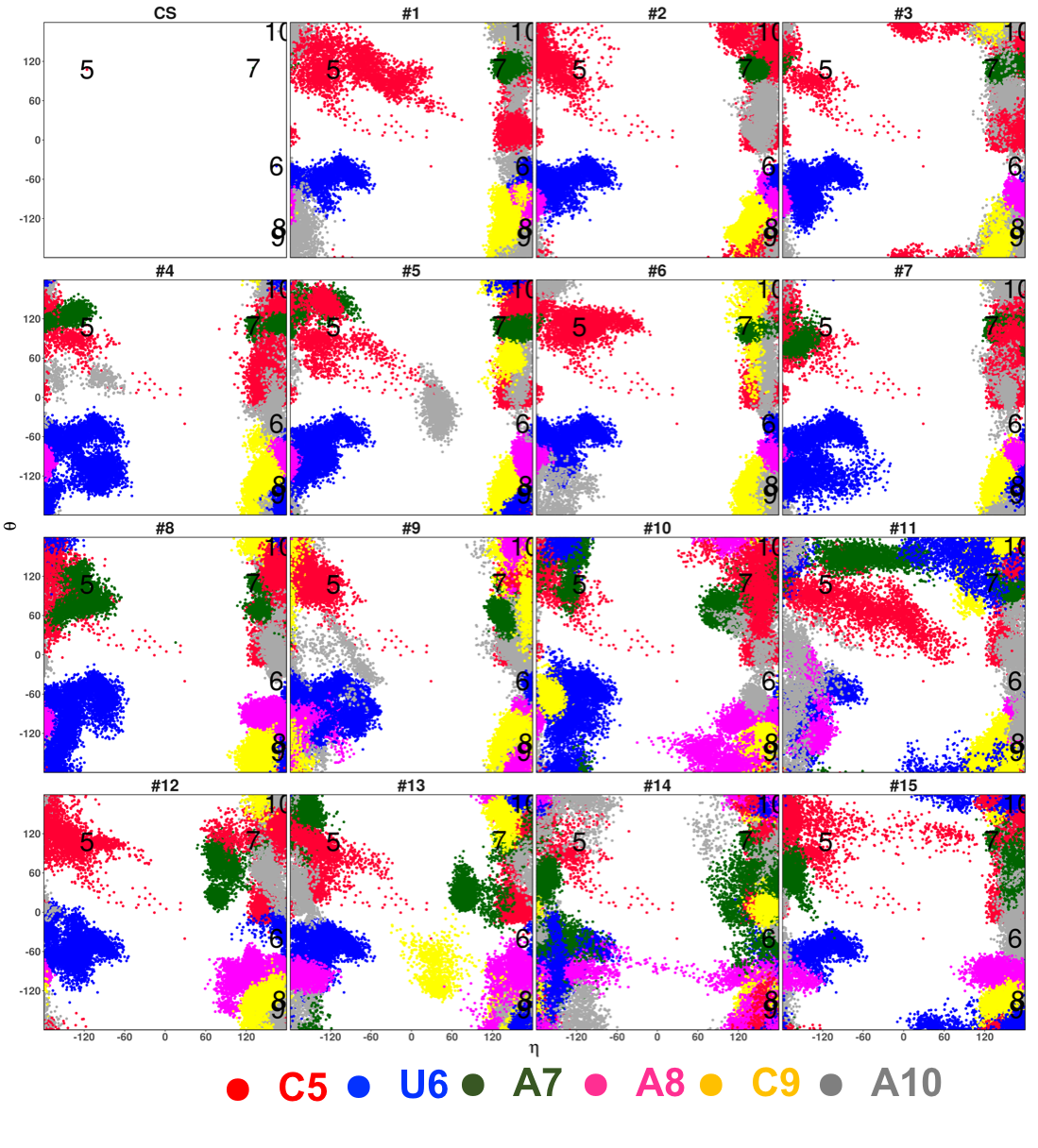


Figure SI9: η-θ plots showing variations of pseudotorsion angles, η and θ for mRNA sampled within each window all umbrella sampling windows as indicated by #N. The first panel (CS) indicates pseudotorsion for mRNA bound to the crystal structure. (pdbid: 4JVH)


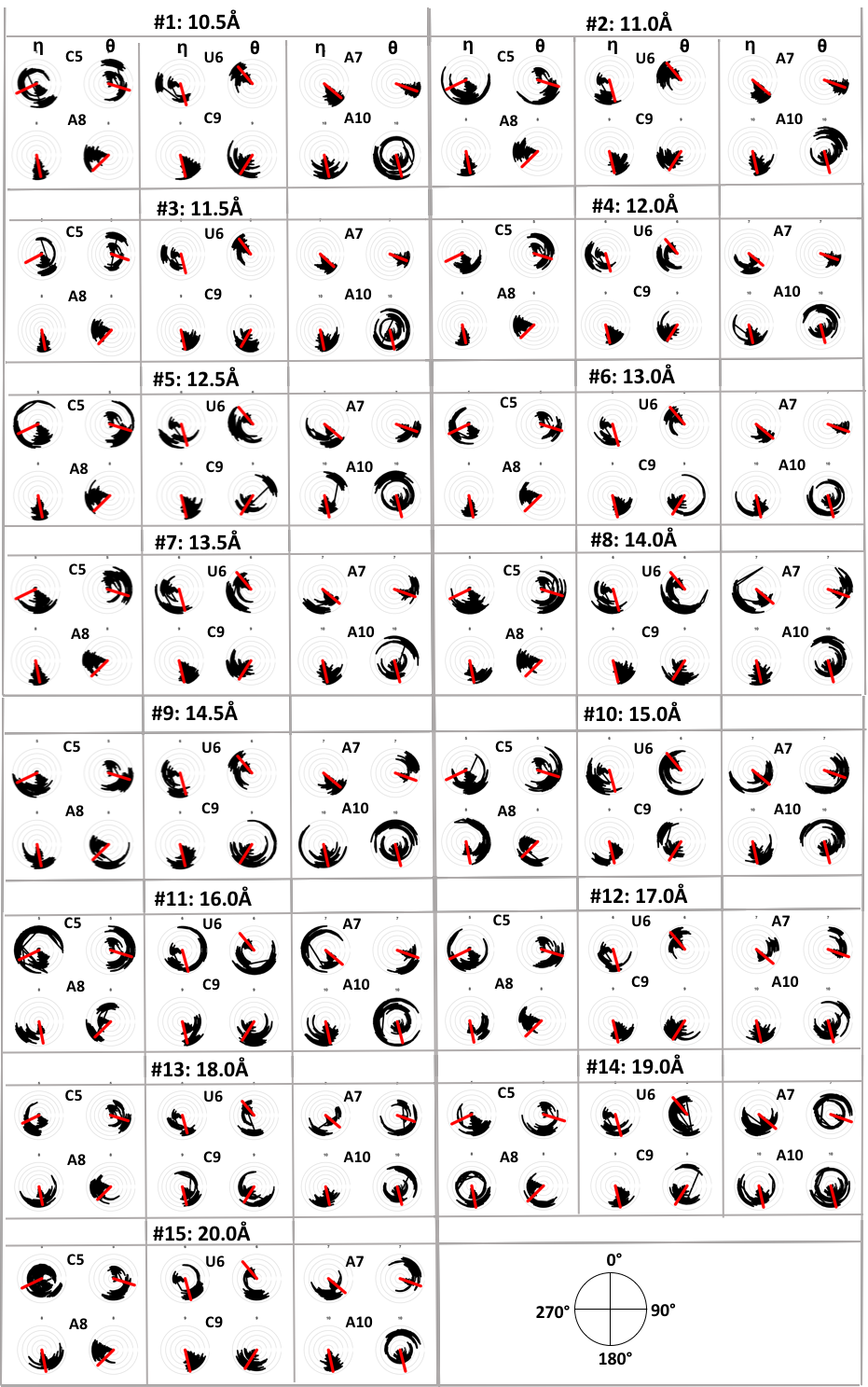


Figure SI10: Dial plots showing variations of pseudotorsion angles, η and θ for mRNA sampled within each window. All umbrella sampling windows and the value of reaction coordinate within each window, is indicated by #N: <Å>. (Red lines denote values for crystal structure conformation (pdbid: 4JVH). Pseudo-torsions are plotted along the polar coordinate system from 0° to 360° as indicated in the last panel and in the circular plots, the radial axis signifies time, with the origin as 0 ps of the production run and progressing outward.
